# Optimal Vaccine Allocation for the Early Mitigation of Pandemic Influenza

**DOI:** 10.1371/journal.pcbi.1002964

**Published:** 2013-03-21

**Authors:** Laura Matrajt, M. Elizabeth Halloran, Ira M. Longini

**Affiliations:** 1 Department of Applied Mathematics, University of Washington, Seattle, Washington, United States of America; 2 Center for Statistics and Quantitative Infectious Diseases, Vaccine and Infectious Disease Division, Fred Hutchinson Cancer Research Center, Seattle, Washington, United States of America; 3 Department of Biostatistics, School of Public Health, University of Washington, Seattle, Washington, United States of America; 4 Emerging Pathogens Institute, University of Florida, Gainesville, Florida, United States of America; 5 Department of Biostatistics, Colleges of Medicine, and Public Health and Health Professions, University of Florida, Gainesville, Florida, United States of America; Imperial College London, United Kingdom

## Abstract

With new cases of avian influenza H5N1 (H5N1AV) arising frequently, the threat of a new influenza pandemic remains a challenge for public health. Several vaccines have been developed specifically targeting H5N1AV, but their production is limited and only a few million doses are readily available. Because there is an important time lag between the emergence of new pandemic strain and the development and distribution of a vaccine, shortage of vaccine is very likely at the beginning of a pandemic. We coupled a mathematical model with a genetic algorithm to optimally and dynamically distribute vaccine in a network of cities, connected by the airline transportation network. By minimizing the illness attack rate (i.e., the percentage of people in the population who become infected and ill), we focus on optimizing vaccine allocation in a network of 16 cities in Southeast Asia when only a few million doses are available. In our base case, we assume the vaccine is well-matched and vaccination occurs 5 to 10 days after the beginning of the epidemic. The effectiveness of all the vaccination strategies drops off as the timing is delayed or the vaccine is less well-matched. Under the best assumptions, optimal vaccination strategies substantially reduced the illness attack rate, with a maximal reduction in the attack rate of 85%. Furthermore, our results suggest that cooperative strategies where the resources are optimally distributed among the cities perform much better than the strategies where the vaccine is equally distributed among the network, yielding an illness attack rate 17% lower. We show that it is possible to significantly mitigate a more global epidemic with limited quantities of vaccine, provided that the vaccination campaign is extremely fast and it occurs within the first weeks of transmission.

## Author Summary

In the past, the emergence of new strains of influenza has been sometimes responsible for large and deadly pandemics. With a very high mortality rate, (i.e., about 60% of the reported cases), H5N1AV influenza, commonly known as bird flu, is thought to be an important potential threat for a new pandemic. Because of this, several vaccines have been developed, but only a few million doses are readily available. Other zoonotic influenza strains, particularly in pigs, also threaten, and vaccines are being produced for them as well. In the event of an influenza pandemic, utilizing these resources optimally could make the difference between dealing with a serious infectious disease at a global scale and reducing it to a highly localized and controlled outbreak. In this paper, we address this issue by developing a mathematical model of influenza transmission on a network of cities. We couple the model with an optimization algorithm to allocate vaccine in time and space through the network. We find that our optimal allocation strategies can mitigate a pandemic, provided that vaccination occurs quickly, within the first weeks of a potential pandemic. In addition, our analysis highlights the importance of cooperative and coordinated vaccine distribution, if we want to mitigate a pandemic.

## Introduction

Highly pathogenic avian influenza A(H5N1AV) emerged in the 1990s in Southeast Asia with new cases arising in different parts of the world [1]. Recent studies have shown that a few mutations can make the H5N1AV influenza virus transmissible in ferrets [2], [3], reminding us that the possibility of a mutated H5N1AV influenza strain capable of infecting humans might not be a remote one. With a very high mortality ratio (i.e., about 60% of the reported cases), the threat of a H5N1AV influenza pandemic remains one of the biggest public health fears. Many pharmaceutical and non-pharmaceutical interventions can be implemented during a pandemic, but vaccination, when available, is the most effective intervention. Several vaccines are being produced specifically for H5N1AV [4] , but their production is still very limited [5]. In the event of a H5N1AV pandemic, utilizing these vaccines optimally at the beginning of transmission could make the difference between reducing transmission to negligible levels or dealing with a deadly infectious disease on a global scale.

In this highly connected world, people travel fast, and new strains of influenza can travel with them. Indeed, the airline transportation network can accelerate the diffusion of new strains of influenza. For example, the pandemic influenza A(H1N1) 2009 (2009H1N1P) was first detected in Mexico in April 2009, and only two weeks later, more than twenty countries reported their first cases of 2009H1N1P, with most of these cases being imported via airline travel [6], [7]. This highlights the necessity of an extremely fast global response to a new strain of pandemic influenza, on the order of days.

**Figure 1:**
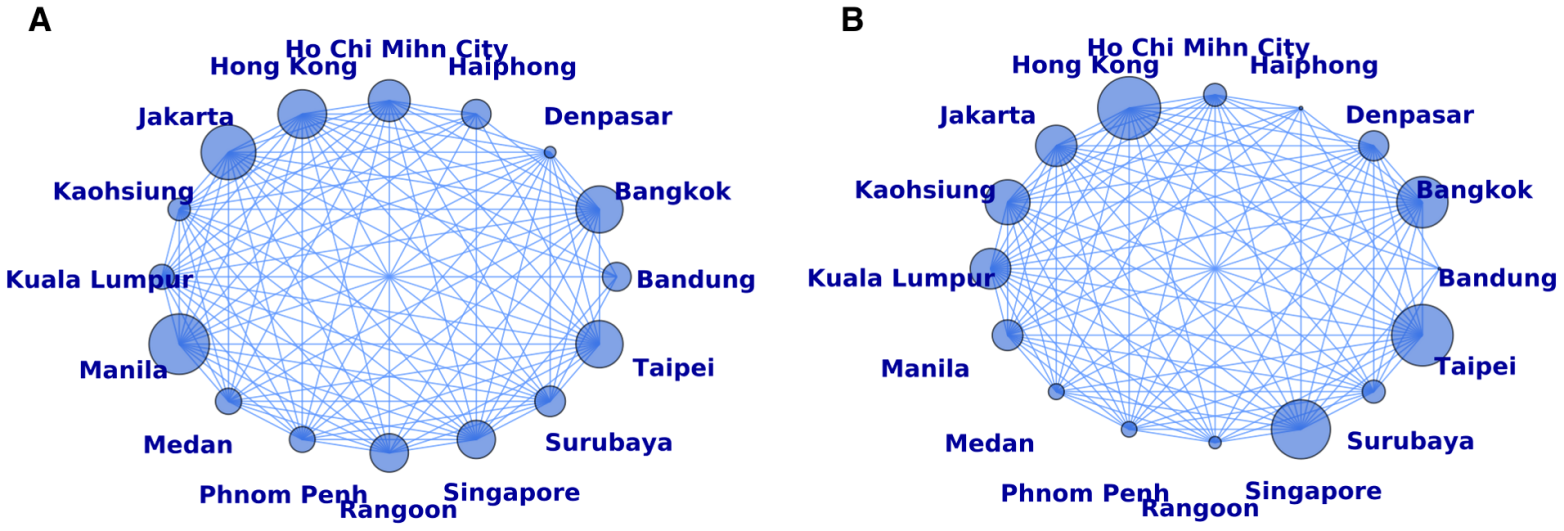
Network representation of 16 cities in Southeast Asia used for the simulations. An edge connecting two cities represents daily travel between those cities. The size of the nodes in (A) correspond to the population size relative to the total population in the network. The size of the nodes in (B) correspond to the flux of passengers traveling through each city relative to the total number of flights in the network. The base case simulations were started in Jakarta, which accounts for 12.8% of the total population of the network but only 7% of the total daily travel goes through it.

Mathematical models are useful tools to explore different pandemic scenarios and possible interventions, and they are particularly well-suited for determining optimal vaccine distribution. With pioneering work starting in the 1970s, [8], [9] and more recently [10], [11], [12], [13], [14], [15], [16], [17], [18], [19], [20], [21], important progress has been made in investigating the optimal resource allocation for a given population. Most of this work has been centered around a single population, and assumes that all the vaccine, enough to cover a significant fraction of the population, will be available prior to an epidemic. In the present work, we focus on optimizing vaccine allocation in a network of cities when only a few million doses are available, and we search for their optimal distribution by minimizing the illness attack rate (i.e., the percentage of people in the population who became infected and ill). We couple a mathematical infection transmission model with two age-groups, children and adults, with a fast genetic algorithm [22] to optimally and dynamically distribute vaccine through a network of cities, connected by the airline transportation network. Since Southeast Asia has seen more than half of the H5N1AV influenza related deaths worldwide, we consider this region for our model. We selected the most highly populated 16 cities in Southeast Asia for which we could find reliable airline transportation data (fig. 1). This represents 10 different countries and a total population of 70,980,365 people (table 1). These cities are highly connected (fig. 1) and were previously shown to form a transmission cluster in the global network of cities [23]. We show that the vaccine allocation proposed by our optimizer could greatly reduce the attack rate.

**Table 1:** Population values.

City	Population	Percentage of the total population	Nation	Percent under 20^a^
Bandung	2,510,982	3.5	Indonesia	38.32
Bangkok	6,704,000	9.4	Thailand	29.95
Denpasar	405,923	0.5	Indonesia	38.32
Haiphong	2,614,764	3.7	Vietnam	35.23
Ho Chi Minh City	5,314,000	7.5	Vietnam	35.23
Hong Kong	7,206,000	10.2	China	19.30
Jakarta	9,125,000	12.9	Indonesia	38.32
Kaohsiung	1,526,575	2.2	Taiwan	22.56
Kuala Lumpur	1,887,674	2.7	Malaysia	41.39
Manila	11,100,000	15.6	Philippines	44.24
Medan	2064719	2.9	Indonesia	38.32
Phnom Penh	2,009,264	2.8	Cambodia	45.78
Rangoon	4,477,638	6.3	Burma	41.52
Singapore	4,436,000	6.3	Singapore	25.65
Surabaya	2,845,000	4.0	Indonesia	38.32
Taipei	6,752,826	9.5	Taiwan	22.56
TOTAL	70,980,365	100		

a The percentages for each city were computed from [58] using the countr y's percentage of children under 20 years old. Taiwan's percentage was obtained from [59].

## Results

### Results for a single batch of vaccine

Because we are interested in investigating the optimal use of vaccine for a quick response, we concentrate most of this work on optimizing vaccine delivered in a single batch at the beginning of the epidemic when only a few million doses are available. We considered four vaccination days very early in transmission, at either 5, 10, 15, or 30 days after its start, and two vaccination days later on at either 60 or 90 days after the beginning of transmission. Since we cannot know how much vaccine production will be ready at the time of an epidemic, for each of these control days, we consider allocating two, four, five, six, seven or ten million doses corresponding to vaccinating 2.8, 5.6, 7.0, 8.5, 9.9 or 14.1% of the total population, respectively (table 2).

**Table 2:** Vaccine coverages considered.

Million doses	Percentage of the total population
2	2.8
4	5.6
5	7.0
6	8.5
7	9.9
10	14.1

For each possible vaccination day and coverage combination, we compare the best vaccine allocation given by the genetic algorithm, denoted as the optimal strategy, to a baseline scenario, where no vaccine is available, and two other possible allocations. The first is the pro rata strategy, where we distribute vaccine to each age-group in each city proportional to the age-group's size. For example, if adults in city 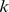 correspond to 20% of the total population in the network, then we assign 20% of the available resources to the adults in city 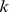. The second strategy is the children-only pro rata strategy, where vaccine is distributed only to the children in each city proportional to the children's population size.

All the simulations presented below started in the same city, Jakarta, with 10 infectious people. Fig. S1 shows the epidemic curves when no vaccination is applied. We also started our simulations in Hong Kong and Taipei. The transmissibility of an infectious disease is often characterized by the basic reproduction number, 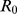, defined as the expected number of secondary infections resulting from a single typical infectious person in a completely susceptible population. In the results presented here, 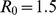, corresponding to a virus roughly as transmissible as the 2009H1N1P [24], [25]. We also considered a less (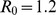) and a more (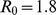) transmissible virus.

**Figure 2:**
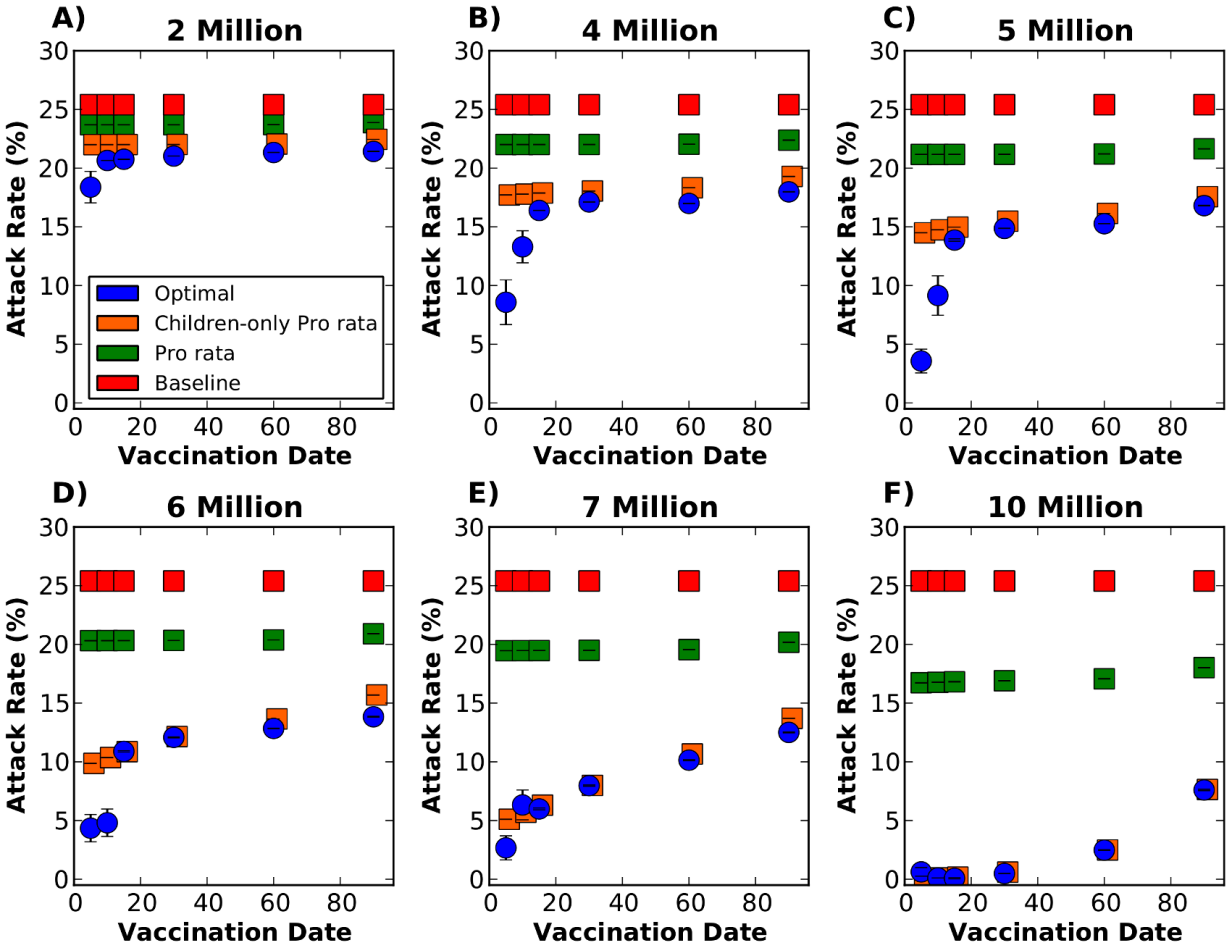
Attack rate with 95% bootstrapped CI with the epidemic starting in Jakarta. This figure shows the results for a single intervention for six different vaccination days considered and six different vaccination coverages. Each panel represents a given number of vaccine doses available to distribute in the entire network: A) Two million doses. B) Four million doses. C) Five million doses. D) Six million doses. E) Seven million doses. F) Ten million doses. For each panel, each point in the graph corresponds to the attack rate for a single vaccination day, either on day 5, 10, 15, 30, 60, or 90 after the beginning of the epidemic. The optimal, pro rata and children-only pro rata strategies are shown in blue, green and orange respectively. The baseline scenario (red) indicates no vaccination. For each vaccination coverage and day combination, the optimal strategy considerably outperformed the pro rata strategy. When vaccination occurs early in the epidemic, and few doses are available, the optimal strategy also outperforms the children-only pro rata strategy.

The optimal strategy shows a modest decrease in the attack rate when very little vaccine is available, provided vaccination occurs during the first five days of the epidemic (i.e., 27% reduction in the attack rate relative to the baseline for two million doses, fig. 2A). As more vaccine becomes available, the optimal strategy greatly reduces the attack rate, although this effect is attenuated as vaccination starts later in the epidemic. For example, if vaccination occurs on day five with five million doses, 3.5% of the population would become infected, with an 85% reduction in the attack rate compared to the baseline case. But if vaccination occurs on day 30, then 14.9% of the population would become infected with only 41% reduction compared to baseline (fig. 2C). Once 10 million doses are available, the optimal strategy can interrupt transmission as long as vaccination occurs before or on day 30 (fig. 2F).

The optimal strategy outperforms the pro rata strategy for all scenarios considered. With only two million doses available, there is a slight difference in the attack rate with early vaccination (5% difference), but this difference tends to disappear as we start vaccination later in the epidemic (fig. 2A). As more vaccine becomes available, this difference becomes more noticeable, peaking when five and 10 million doses are available (17% and 16% difference in the attack rate, respectively, fig. 2C and 2F). Our results suggest that for the vaccine coverages considered, the pro rata strategy is somewhat insensitive to the timing of the intervention, while the other two strategies considered are not. This would further imply that the pro rata strategy has little indirect effects of herd immunity, but still protects the individuals being vaccinated.

When compared to the children-only pro rata strategy, the optimal strategy performs better only when vaccination occurs early in the epidemic and there are few doses of vaccine available (fig. 2B–D). As more vaccine becomes available, the optimal strategy and the children-only pro rata strategy yield very similar attack rates, and for some cases the optimal strategy is in fact the children-only pro rata strategy (for example fig. 2F, vaccination on day 30, 60 or 90).

**Figure 3:**
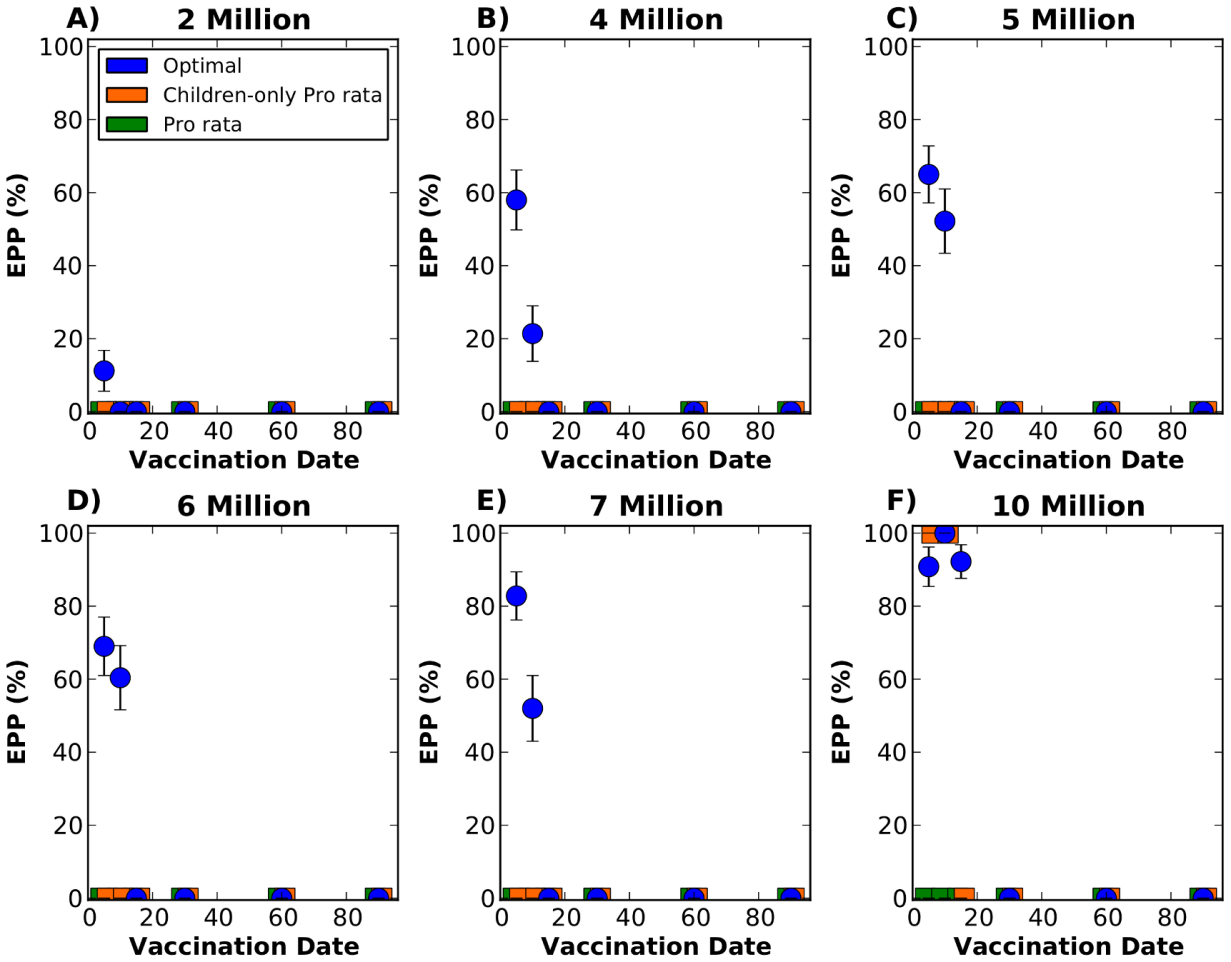
Epidemic prevention potential (EPP) with 95% bootstrapped CI with the epidemic starting in Jakarta. Each panel represents a given number of vaccine doses available to distribute in the network. A) Two million doses. B) Four million doses. C) Five million doses. D) Six million doses. E) Seven million doses. F) Ten million doses. Each point in each graph corresponds to the EPP for a single vaccination day, either on day 5, 10, 15, 30, 60, or 90 after the beginning of the epidemic. The optimal, pro rata and children-only pro rata strategies are shown in blue, green and orange respectively. When fewer than 10 million doses of vaccine are available, only the optimal strategy is capable of mitigating a significant fraction of the epidemics if vaccination starts early. With as few as 4 million doses, the optimal strategy can mitigate as many as 57% of the epidemics.

We next investigate the capacity of a strategy to mitigate a large epidemic (where more than 1% of the population in each city got infected and ill) from occurring. To this end, we calculate for each solution the epidemic prevention potential (EPP) [26], defined as one minus the ratio of the probability of an epidemic given a particular intervention over the probability of an epidemic given no intervention (fig. 3). Mathematically,
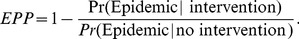
(1)


The optimal strategy is able to mitigate epidemics with very low quantities of vaccine, provided that vaccination starts very early in the epidemic. With as little as four million doses, the optimal strategy mitigates 58% of the epidemics if vaccination starts on day 5, but only 21% if vaccination starts on day 10 (fig. 3B). As more vaccine becomes available, the optimal strategy is able to mitigate a higher proportion of epidemics (fig. 3C–F). In contrast, the other two strategies considered fail to mitigate the epidemics in all of the scenarios considered when less than 10 million doses are available.With 10 million doses, the optimal strategy mitigates over 90% of the epidemics as long as vaccination occurs during the first 15 days. If vaccination occurs during the first ten days, children-only pro rata is also able to mitigate the majority of the epidemics with ten million doses.

**Figure 4:**
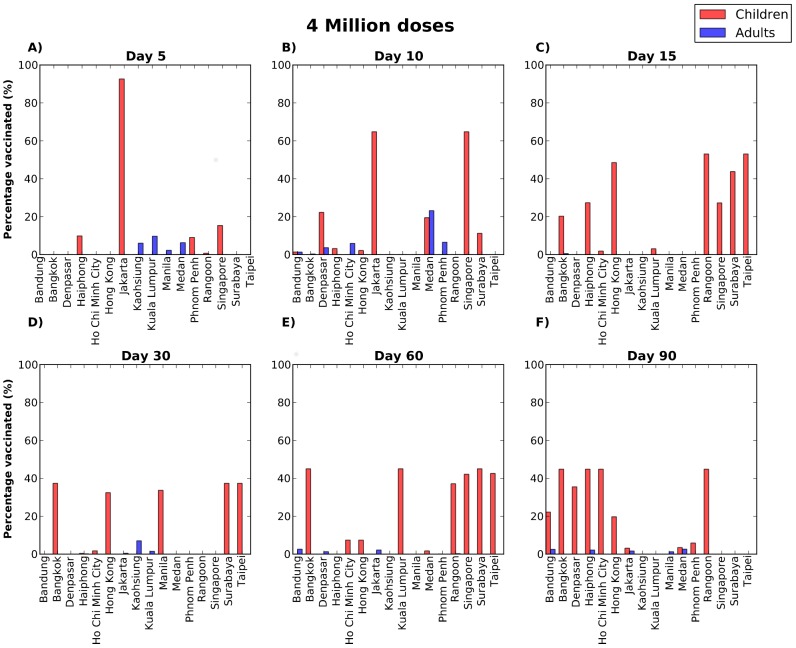
Optimal vaccine distribution when four million doses are available and the epidemic is started in Jakarta. Each panel corresponds to allocating vaccine on a different day: either 5 days (A), 10 days (B), 15 days (C), 30 days (D), 60 days (E), or 90 days (F) after the beginning of the epidemic. Each bar corresponds to the percentage of children (red) or adults (blue) vaccinated in each city. These results suggest that the geographical allocation of vaccine is important early in the beginning of the epidemic, when the optimal strategy allocates most of vaccine in Jakarta, but then it is better to distribute vaccine evenly among children up to a certain threshold in time when it becomes important to allocate the vaccine to those cities where either the epidemic has not peaked yet or it is possible to reach a higher proportion of children vaccinated.

**Figure 5:**
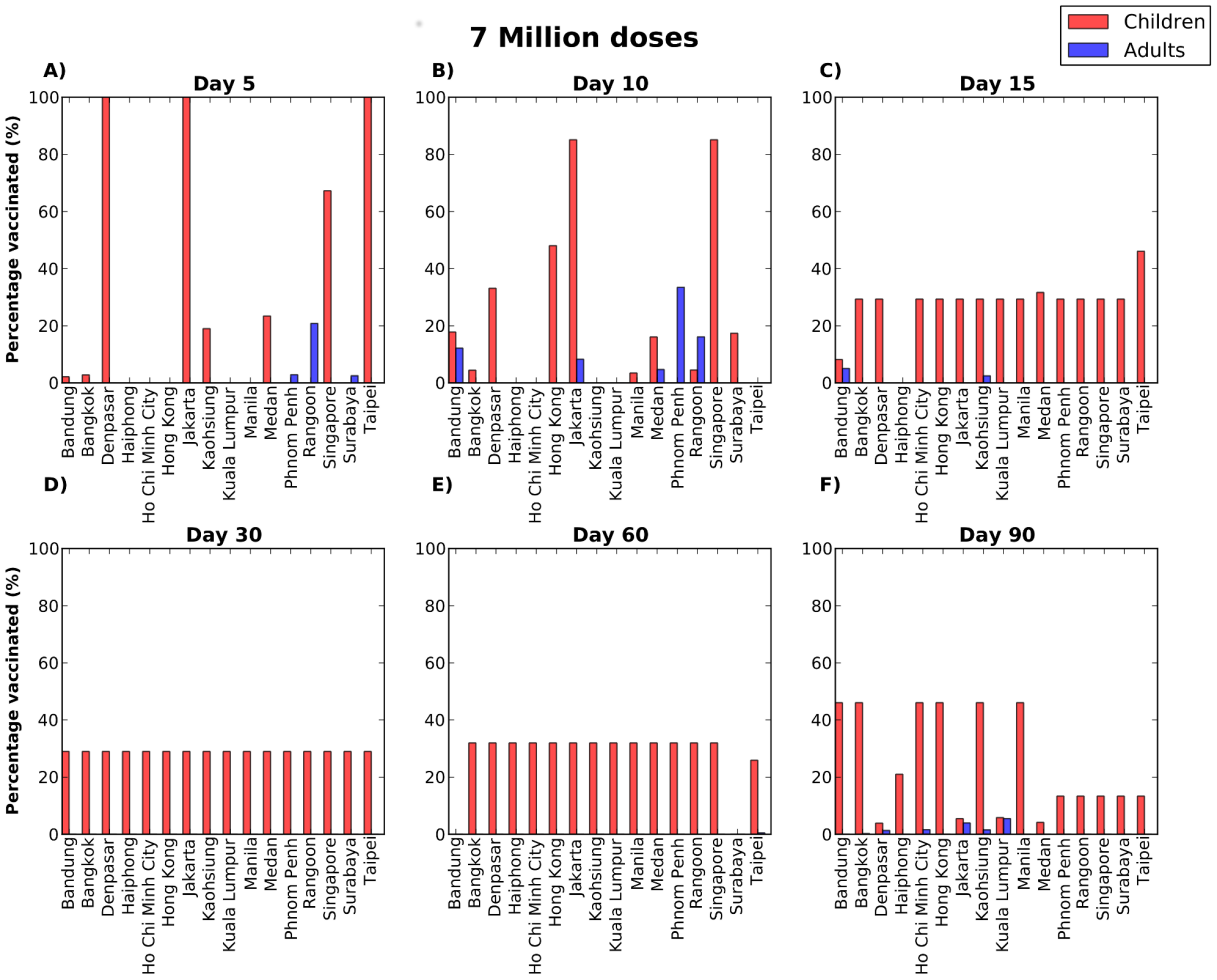
Optimal vaccine distribution when seven million doses are available and the epidemic is started in Jakarta. Each panel corresponds to allocating vaccine on a different day: either 5 days (A), 10 days (B), 15 days (C), 30 days (D), 60 days (E), or 90 days (F) after the beginning of the epidemic. Each bar corresponds to the percentage of children (red) or adults (blue) vaccinated in each city. When vaccination occurs early in the epidemic, the optimal strategy is to allocate most of the available vaccine in Jakarta. As vaccination occurs later on, the optimal strategy switches to favor a more evenly distribution of vaccine amongst children. For late vaccination, allocating vaccine evenly in children becomes less relevant but instead the optimal strategy allocates vaccine to fewer cities where the epidemic has not peaked yet or it is possible to reach a high proportion of children vaccinated.

Our results suggest that with limited quantities of vaccine, the geographical allocation of vaccine is key in stopping the epidemic if vaccination occurs early on, with most of the vaccine going only to a few cities (figs. S2A, S3A). However, as the epidemic progresses, or more vaccine becomes available, allocating resources more evenly, to the high transmission groups, here the children, becomes a predominant feature (figs. 4C–E and 5C–E). For late vaccination, allocating vaccine in children becomes less relevant and again, geographical location is more important, with the optimal strategy favoring cities where either where the epidemic has not peaked yet or it is possible to reach a high proportion of children vaccinated (figs. 4F, 5F). This is in agreement with previous work [27], [28], [20], which have suggested that vaccinating children is important early in the epidemic, but that there is a threshold after which other groups might benefit more from limited quantities of vaccine.

### Sensitivity analysis

When the epidemic starts in Hong Kong, applying the optimal strategy results in a lower EPP and a lower reduction in the attack rate compared to baseline, specially noticeable when only a few million doses of vaccine are available (fig. S2 and S3). Similar results were obtained when the epidemic started in Taipei (figs. S4 and S5). Only 7% of the daily travel through the network goes through Jakarta. In contrast, 17% of the daily flux of travelers occurs (fig. 1B and table 3) in Hong Kong (16% of the daily flux occurs in Taipei). Our results suggest that if an epidemic starts in a city with a more important flux of travelers, it will spread more rapidly and will be more difficult to mitigate.

**Table 3:** Daily flux of passengers through the network.

City	Percentage of the total travel
Bandung	0.03
Bangkok	11.52
Denpasar	3.95
Haiphong	0.05
Ho Chi Minh	2.31
Hong Kong	17.36
Jakarta	7.47
Kaohsiung	8.85
Kuala Lumpur	7.23
Manila	4.13
Medan	1.1
Phnom Penh	1.06
Rangoon	0.66
Singapore	15.33
Surabaya	2.32
Taipei	16.61

Percentage of the total number of daily passengers traveling through each city considered in the network.

Our results were not sensitive to changes in the basic reproductive number. When the epidemic is less transmissible, all three strategies perform better, with the optimal strategy being slightly better than the children-only pro rata, both yielding to substantial reductions in the attack rate (fig. S6). This is expected, as we know from previous studies [29] that for a low 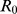 as this one, the threshold in deterministic models for the number of children and adults needed to be vaccinated to bring 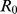 below 1 is much lower. The optimal strategy could mitigate most of the epidemics with as little as two million doses of vaccine (fig. S7). For a higher 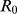, the optimal strategy still outperforms the pro rata strategy. If vaccination occurs early on, this difference is more prominent than for 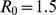, suggesting that when the virus is more transmissible allocating the resources optimally is more important, at least at the beginning of the epidemic (see Text S1 for details).

Our model assumed that infectious symptomatic individuals will reduce their probability of traveling by 25%. We performed sensitivity analysis with respect to this parameter (fig. S10 and S11). We also performed sensitivity analysis with respect to the reduction of the travel probability of an infected child compared to the travel probability of an infected adult (fig. S12). As expected, if more infected symptomatic people travel through the network (10% reduction in the probability of travel) the attack rates are slightly higher (fig. S10A), and the EPP is not as good (fig. S11A). This makes sense since in this case the epidemic process is accelerated. On the other hand, if the reduction in the travel probability is higher (75% reduction) then less infected symptomatic people are traveling through the network. Here, the attack rates are lower and the optimal strategy can prevent epidemics for more days (figs. S10B, S11B, and S12). Our conclusions were not sensitive to changes in the probability of travel for symptomatic infectious people (both adults and children), or in changes in the probability of travel for symptomatic infectious children in the sense that the optimal strategy outperformed the other two strategies considered. The biggest difference was still when vaccination occurred during the first days of the epidemic (see Text S1).

We also varied the values of the vaccine efficacies. We considered a poorly-matched vaccine (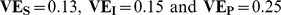) and a moderately-matched vaccine (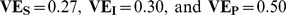). These values correspond to one-third and two-thirds of the values used for the original analysis, which assumed that the vaccine would be as efficacious as seasonal vaccines. As expected, if the vaccine is poorly-matched, all the strategies perform poorly yielding high attack rates (fig. S13A), and all of them fail to prevent the epidemics (fig. S14A). If the vaccine is moderately-matched, the results are close to the original results, with the optimal strategy yielding clear reductions in the attack rates (fig. S13B) and a significant proportion of the epidemics prevented, when vaccination occurs early on (fig. S14B). Furthermore, we performed sensitivity analysis to the assumption that vaccine is administered at once, and repeated the optimization assuming that the vaccination would be completed in 10 days (fig. S15, see Text S1 for details). While the optimal strategy still performs better than the other strategies considered, the difference in the attack rate (panel A) is not as marked as before. The optimal strategy in this case could prevent 36% of the epidemics but only if vaccination started on day 5 (panel B).

Finally, we performed the optimization assuming that only children could receive vaccine. Here, the optimal strategy also outperforms the other two strategies considered (fig. S16A), and yields an EPP similar to the one obtained when the optimization was performed using the entire population (fig. S16B, see Text S1 for details). These results suggest that there might be a tradeoff between vaccinating specific geographical locations and vaccinating high-transmission groups. For some vaccine coverages or vaccination dates, it might be better to vaccinate children and adults in a city to guarantee that the reproduction number is below 1 in as many cities as possible, but for other coverages or dates it might be better to concentrate the effort in the children (the high-transmission group). This is consistent with previous work [30], [12], [27], [31], [32], [33], which have proposed tradeoffs between vaccinating the high-transmission groups and the high-risk groups.

### Results for two batches of vaccine

**Figure 6:**
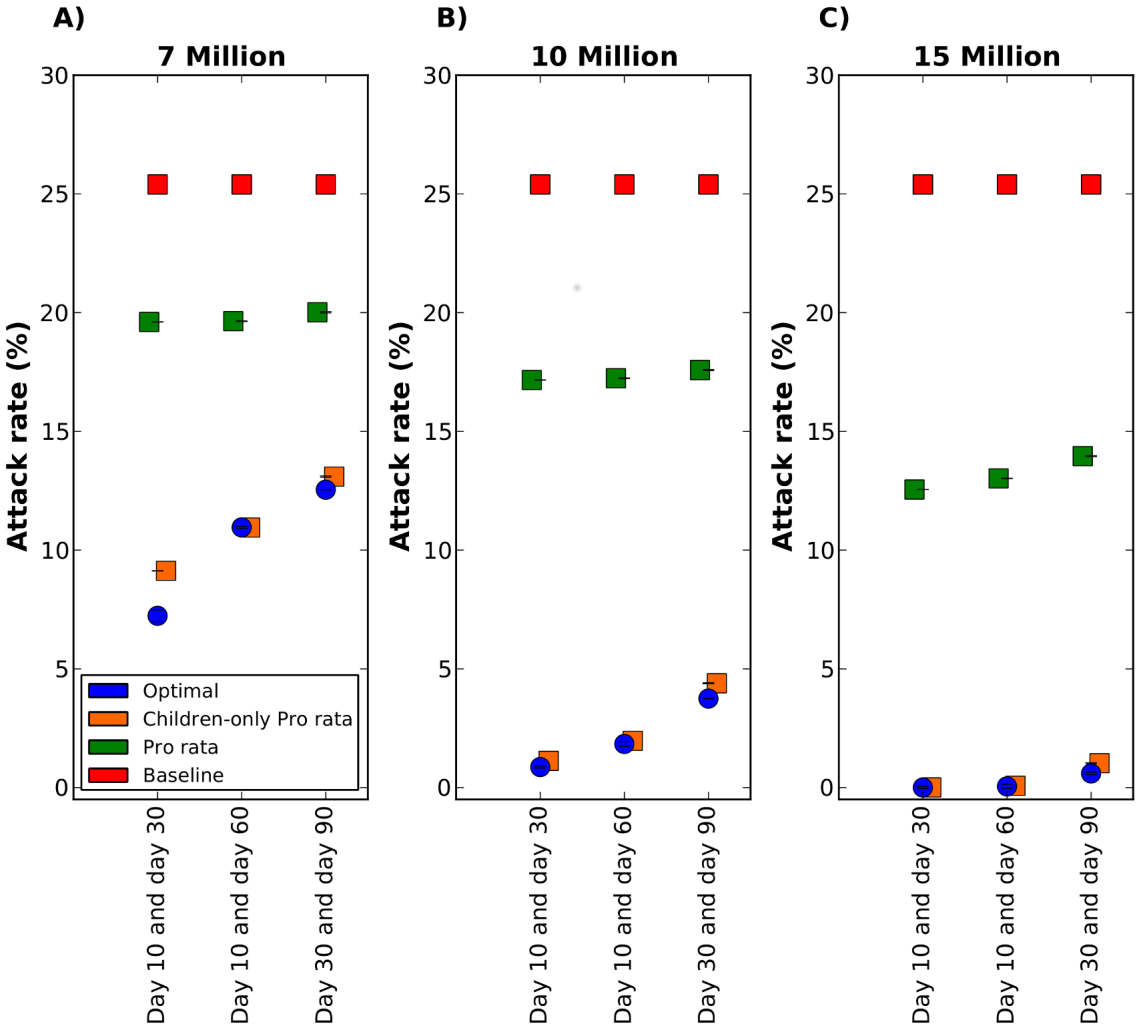
Attack rate for vaccine allocation in two batches on two different days. Three vaccine coverages are considered: A) Seven million doses of vaccine total, with two million available on the first day and five million available on the second day. B) 10 million doses of vaccine total, with five million doses of vaccine available on each day. C) 15 million doses of vaccine total, five million doses available on the first day and 10 million doses available on the second day. In each panel, three combinations of vaccination days are considered: vaccination on day 10 and day 30, day 10 and day 60, or day 30 and day 90. Here, the epidemic was seeded in Jakarta. The optimal, pro rata and children-only pro rata strategies are shown in blue, green and orange respectively. Here, the optimal strategy and the children-only pro rata strategy yield similar attack rates for all scenarios considered except for one. When seven million doses of vaccine are available at days 10 and 30, the optimal strategy yield a slightly lower attack rate than the children-only pro rata strategy.

In this section we present the results when vaccine is considered to be available in two batches, the first one carrying only two or five million doses and the second one with either five, 10, or 15 million doses. We further consider allocating vaccine on either days 10 and 30, days 10 and 60, or days 30 and 90 after the beginning of the epidemic. Here, the optimal strategy basically coincides with the children-only pro rata strategy for all scenarios considered but one, where the optimal strategy has a modest 2% lower attack rate (Fig. 6A). For all the scenarios considered, these strategies perform much better than the pro rata strategy, particularly when vaccination occurs on days 10 and 30, with a maximal reduction of 17% in the attack rate (Fig. 6B).

**Figure 7:**
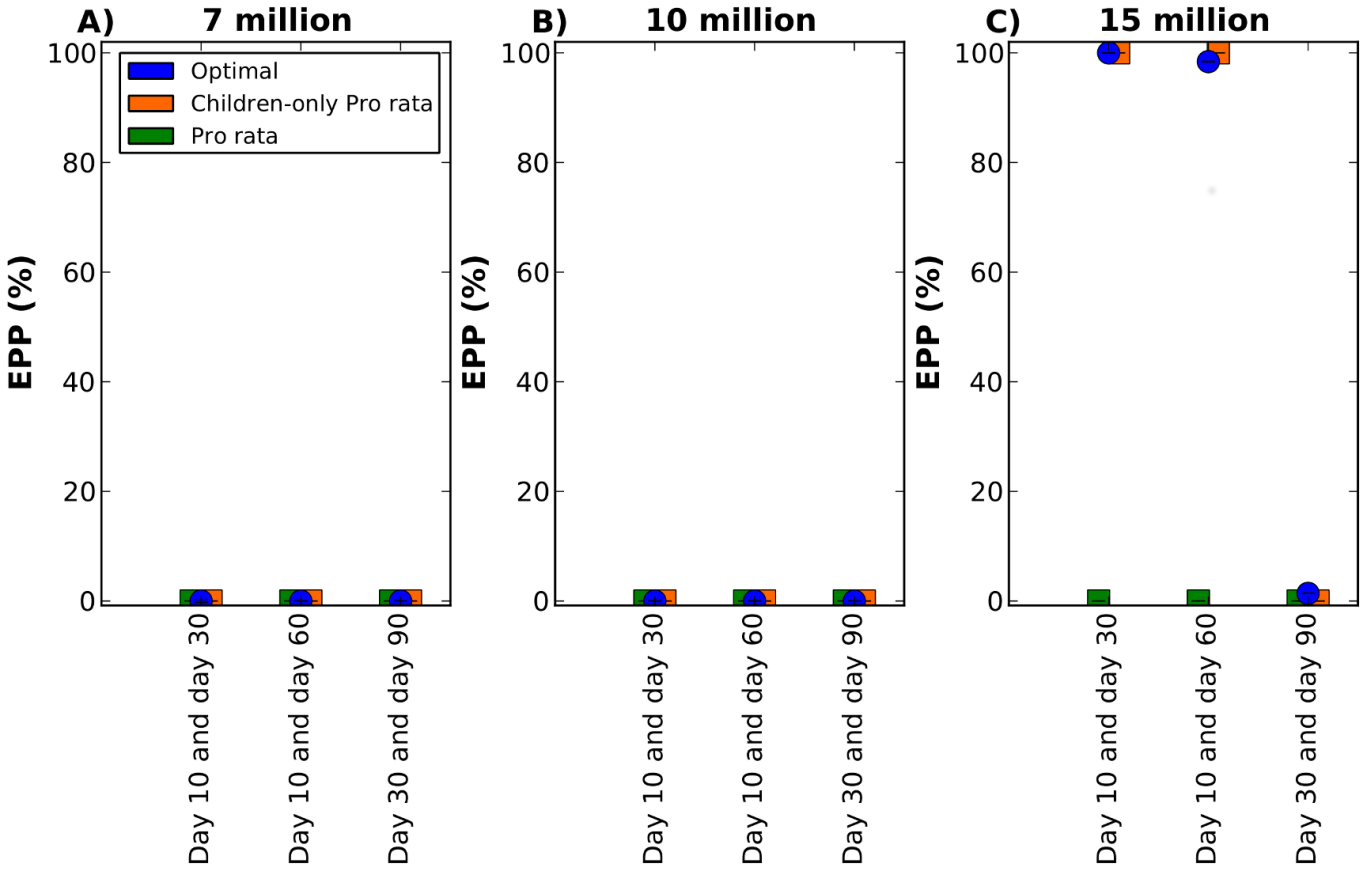
Epidemic prevention potential for vaccine allocation in two batches on two days. Three vaccine coverages are considered: A) Seven million doses of vaccine total, with two million available on the first day and five million available on the second day. B) 10 million doses of vaccine total, with five million doses of vaccine available on each day. C) 15 million doses of vaccine total, five million doses available on the first day and 10 million doses available on the second day. In each panel, three combinations of vaccination days are considered: vaccination on day 10 and day 30, day 10 and day 60, or day 30 and day 90. The optimal, pro rata and children-only pro rata strategies are shown in blue, green and orange respectively. Here, the epidemic was seeded in Jakarta. All strategies fail to mitigate the epidemics if seven or 10 million doses are available in two batches. With 15 million doses, the optimal strategy and the children-only pro rata strategy mitigate over 95% of the epidemics when the first batch of vaccine is delivered on day 10 and the second one is delivered either on day 30 or on day 60.

With seven or ten million doses (both batches included), all the strategies considered fail to mitigate the epidemic (Fig. 7A–B). With 15 million doses of vaccine, the optimal strategy and the children-only pro rata strategy mitigate over 95% of the epidemics if vaccination occurs at days 10 and 30 or 10 and 60, but cannot mitigate any epidemic if vaccination occurs at days 30 and 90 (Fig. 7C). These results are expected, since we found that for a single intervention, the EPP was zero for all the strategies considered if vaccination started after day 30.

## Discussion

We propose here a mathematical model of influenza transmission coupled with a fast genetic algorithm, which provides strategies for vaccine allocation in a network of cities, rather than in an individual population. Our results show that the optimal strategy found by our genetic algorithm always outperforms a pro rata strategy, both in reducing the total final attack rate and in increasing the probability of mitigating an epidemic, with differences in the attack rates as high as 16% of the population.

This conclusion is consistent with previous findings [34]. Substantial study has been done to optimally allocate vaccine within a country or a population [11], [12], [8], [9], [15], [16], [17], [18], [19], [20], [35], [21]. Furthermore, significant advances have been made in optimizing resources for an epidemic in a network: Wu *et al.* considered a metapopulation model of the continental US in 2007, and they compared vaccine allocation in a prorated schedule versus other types of optimal policies [34]. Dimitrov *et al.* optimized antiviral allocation for the 2009 influenza epidemic in the United States [11]. More recently, Klepac and colleagues [36] developed a model of two coupled populations to incorporate economic costs in finding optimal vaccination thresholds. Keeling and Shattock [13] used the final epidemic size to compute optimal vaccine distribution for two interacting communities. The present study contributes to the body of knowledge by incorporating stochastic components in the model and by allowing the possibility of dynamic allocation of resources, both before and after the beginning of an epidemic. In addition, our work can easily be adapted for other infectious diseases. For example, our methodology could be used for the novel reassortant influenza A(H3N2) virus, originating from swine, avian, and human viruses which has been spreading from swine to humans, and then among humans in the United States [37]. Indeed, because wider spread of this virus is possible, plans are underway to make limited quantities of this vaccine if needed.

Furthermore, the results presented here emphasize the importance of confronting a global problem with a global solution, rather than many individualistic ones. Indeed, when little vaccine is available, sharing resources and optimally vaccinating early in the outbreak results in mitigating the epidemic most of the time, while the pro rata strategy always results in an epidemic with high attack rates. This is in agreement with previous work by Colizza *et al.* [38], in which they compared three different strategies for antiviral use and found that cooperative strategies perform better than non cooperative strategies. Our study also highlights the importance of having a good global surveillance system. In our results, most of the epidemics could be mitigated with as little as five million doses of vaccine provided that we act fast, during the first days of an epidemic, but no mitigation is likely to succeed after just a few weeks (30 days). In brief, our results suggest that a global stockpile of vaccine as the one established by WHO [39] would be fundamental to stop an H5N1AV influenza pandemic.

The framework presented here has several limitations. Once the epidemic starts in a city, our model becomes deterministic, and this comes with simplifying assumptions. While in the present study the objective function was set to minimize the overall illness attack rate, the optimal solution depends heavily on attaining the critical vaccination coverage in each city: indeed, under the deterministic paradigm, an epidemic will not take off in a city if 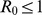. This threshold will be achieved once a significant fraction of children is vaccinated [29]. In reality, even if 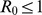, a small epidemic exceeding the 1% threshold we set for EPP could possibly occur. Thus our EPP values could be overly optimistic. The network used here is a closed network, which does not allow for any immigration or emigration of passengers from other cities and we did not consider any flow of passengers through ground transportation. Any epidemic will probably spread beyond this network, potentially affecting the effectiveness our results. The beginning of an epidemic in each city was approximated by a simple birth-death process, and it does not differentiate between the number of infected children and the number of infected adults present in the city. We could not find reliable data regarding the probability of an infected person suspending a trip due to illness, or differential rates for traveling stratified by age. In this sense, our model is conservative. Better data is urgently needed to create more realistic models. Clearly, the simultaneous use of multiple interventions would be more efficient in controlling an epidemic, so an optimization routine that considers a portfolio of interventions points the direction for future work. We assumed that the vaccines for H5N1AV influenza virus would be as efficacious as the seasonal vaccines. However, the current available vaccines for avian influenza might not be well-matched to a new avian influenza virus. Clearly, the effectiveness of any vaccination policy relies on the efficacy of the vaccine used. Indeed, if the vaccine were poorly-matched, then the optimal solution, while still being better than the other two strategies considered, would not be very efficacious. This highlights the necessity to recognize the influenza strain quickly, so that the efficacy of the vaccine can be rapidly evaluated, and additional targeted mitigation measures can be implemented if necessary. In addition, our model does not account for antibody buildup. Adding this feature would probably yield results similar to those when vaccine is completed in several days. Our sensitivity analysis showed that the attack rates and the EPP were considerably lower when vaccination was completed in several days. This highlights the importance of a fast and aggressive vaccination campaign. In addition, our results were sensitive to the knowledge of the exact point in the epidemic (calculated starting from the introduction of the first infected individuals) when vaccination was applied, specially during the first days of an epidemic. This could become problematic in a real situation, since inferring the exact date of the beginning of an epidemic from epidemiological data might be very difficult to achieve. The comparison between one and two batches of vaccine showed that the results were sensitive to the assumption of an a-priori knowledge of the vaccination times. As the H1N1 2009 influenza epidemic showed us, different problems can arise during the production of a vaccine leading to delays in its delivery. In this sense, our conclusions are optimistic. The results presented here were obtained using a genetic algorithm. While genetic algorithms have the advantage of being fast and adaptable, they are not guaranteed to converge. We have reported here our best solutions and shown that even if these solutions are only nearly optimal, they still perform much better than the status quo strategies, which are usually pro rata.

Many of the current governmental or institutional guidelines for vaccine allocation are indeed based in a prorated type strategy, where vaccine is distributed to states or countries according to their population (e.g [40], [41]). While this is presumably the most fair strategy, it could also be, depending on the objective function used, not the optimal use of resources. Optimal strategies depend heavily in the objective function used, and different objective functions can give rise to contradicting vaccination policies [42], [13], [34]. While our optimal strategy significantly reduces the overall attack rate, it creates an inequitable distribution of burden of disease, with some cities having no epidemic at all and others experiencing a big epidemic. An objective function with constraints in the distribution of vaccine would be more fair. Also, other objective functions could be used instead. For example, one could set the objective function that allocates vaccine in fewer populations but guarantees to attain critical coverage in them, so that 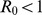, or one could set an objective function that maximizes the EPP, thereby maximizing the probability of a strategy to mitigate an epidemic. Our results suggest that vaccinating only children in a pro rated fashion can be a very efficient solution, and it is more likely to be accepted by the population. A strategy for prioritizing high-risk people and health workers together with children might be a good starting point for discussion. Choosing a vaccination strategy is inherently a difficult process, as one needs to balance concepts like ethics, equality or fairness, together with practical and logistical implications, and economic considerations. We hope to provide decision-makers with the tools to find optimal resource distribution, so that once the goals are established, the available resources can be used at their best.

## Methods

### Mathematical model

We developed a semi-discrete model [43] of a network of 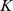 cities connected by means of the airline transportation network, similar to [11]. Namely, the model in each city is a continuous compartmental deterministic model, but we stochastically pulse the populations in the model every day to account for the travel between the cities and to determine if an epidemic will start or not in a susceptible city. We assumed that the most important stochastic effect during an epidemic was the transportation of newly infected people between cities and on the initialization of an epidemic in a city. We then assumed a deterministic course of the epidemic once established in a city.

Table 4 summarizes the parameter values used in the model. The base case scenario had an epidemic with a basic reproduction number 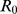 of 1.5 on average. We assumed that vaccine efficacies would be similar to those for the seasonal influenza vaccine, and used the estimates given in [44]. The population in each city is divided into children (age 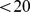 years) or adults (age 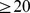 years), where the proportion of people in each age-group for each city is given by the proportion of people in that age-group for that country (see table 1). The contact rates were computed based on [45]. The data for the airline transportation network was taken from [46] and [23]. A full description of the implementation can be found in the Text S1.

**Table 4:** Parameter values.

Parameter	Description	Value	Reference
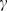	recovery rate	0.25	[60]
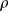	fraction of symptomatic	2/3	[60]
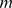	reduction of infectiousness for asymptomatics	0.5	[60]
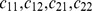	contact rates	1,0.1155, 0.1155, 0.4744	calculated.
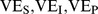	vaccine efficacies for susceptibility, infectiousness and pathogenicity	0.4, 0.45, 0.75	[44]
p	probability of transmission	0.4527	calculated^a^

a This probability of transmission gives rise to a basic reproduction number of *R*_0_ = 1.5.

Our model is written in Python 2.7 (http://www.python.org) and Cython (http://cython.org, [47]) using the modules of Scipy and Numpy [48]. This allowed us to use a fast genetic algorithm module written for Python, Pyevolve [22], to find nearly optimal solutions to this problem.

#### Stochastic importation of infectious people

Let 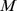 be a matrix representing the airline transportation network (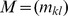), where 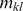 represents the mean number of travelers per day going from city 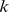 to city 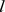. Define 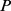 to be a matrix of travel probabilities (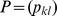), where 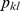 is defined as the probability that a person in city 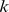 will travel to city 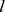. We compute 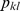 from 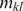 as
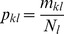
(2)where 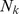 is the total population in city 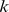, with 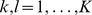.

Each day, a random number 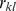 of infected travelers going from city 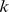 to city 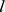 is computed for each of the eight infected classes (children or adults, asymptomatic vaccinated or unvaccinated, symptomatic vaccinated or unvaccinated). For the asymptomatic classes,
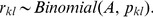
(3)where 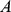 represents the number (rounded to the nearest integer) of infectious asymptomatic people in that class (children or adults, vaccinated or unvaccinated) in city 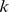 on day 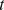 and 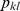 was computed above. The number of symptomatic infectious travelers is computed in a similar way, but the probability is reduced by 25%. Infectious people travel to a new city and stay there for a period of six days, after which they are assumed to be recovered and return to their original city.

#### Stochastic initialization of an epidemic

Let 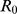 be the basic reproduction number, that is, the expected number of secondary new infections that a single typical infected individual would produce in a completely susceptible population. Let 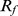 be the effective reproduction number, that is, the expected number of secondary infections that a single typical infected individual would produce in a population where a fraction 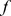 of the population is vaccinated and no natural infection has yet occurred. Each day, the model computes either 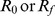 for each city where the epidemic has not started, using the approach given in [49] and [50], [51]. The basic reproduction number will be computed if no vaccine has been distributed to that particular city, whereas the effective reproduction number will be computed when a fraction of the population was previously vaccinated. We approximate the beginning of the epidemic in each city by a standard birth-death process [52], and hence compute the probability 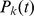 of an epidemic in city 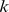 starting on day 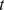 to be
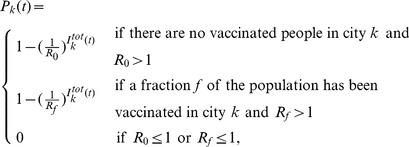
(4)where 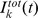 represents the total number of imported infectious people (both symptomatic and asymptomatic) present in city 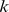 on day 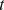. We repeat this process for each city until an epidemic starts in this city (or the end of the simulation is reached).

#### Transmission within cities

Once an epidemic has started in a city, it follows the dynamics given by a deterministic compartmental model developed in [29]. Specifically, the population in each city is divided into two age-groups, children and adults. The population in each city is further divided in susceptibles, infectious asymptomatic, infectious symptomatic, and recovered. Members of each class can be vaccinated or unvaccinated. A full description of the deterministic model can be found in the Text S1.

#### Vaccination

Vaccine distribution to each city and age-group is dictated by the optimizer. Because it is difficult to track susceptible and infectious individuals, especially given that a fraction of the latter are asymptomatic, we assume that only a fraction of the vaccine given to a particular group is used, and we consider the rest of it to be wasted. So if we have 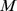 doses of vaccine available for age-group 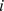 in city 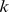, only a fraction of these will be delivered to the susceptible individuals in that group (see Text S1 for details). The vaccine is assumed to be delivered all at once, in a single day. We also assume that vaccinated people are immediately protected.

#### Complete model

A run of the model consists of simulating an epidemic over the network of cities for 250 days. All cities are assumed to have different populations and different percentages of children, but the same contact rate matrix and the same basic parameters for the influenza transmission. Every day, for each susceptible city (i.e a city where no epidemic has started yet), the model counts the number of imported infections in the city and determines if a new epidemic will start or not, using the probability 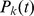, eq. (4). If it does start a new epidemic, it does so with the number of imported infections as initial conditions for the deterministic model. If an epidemic has already started in the city, the model still exports and imports infectious travelers, effectively changing the initial conditions of the deterministic model for the next day. The optimizer predetermines the cities where vaccine will be applied, as well as the quantity of vaccine to be given to a particular city and age-group. If the given day is a vaccination day, then the susceptible populations in those cities are vaccinated as described in the previous section.

### Optimization

We consider the following optimization problem: given limited quantities of vaccine available at given times, what is the optimal vaccine distribution such that the final illness attack rate is minimized?

Formally, suppose 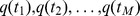 doses of vaccine are available at control times 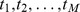. Both the control times and the quantities of vaccine available at each control time are known in advance. Recall that we are considering a network of 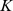 cities, and let 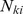 represent the children (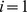) or adults (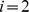) in city 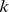. Define a control vector 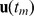 to be a vector in 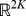,
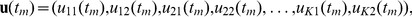
(5)where 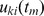 represents the fraction of the population 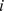 to be vaccinated in city 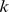 at control time 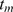. A solution 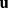 to the optimization problem is a vector of control vectors, each of these corresponding to a given control time,
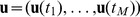
(6)such that 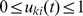 for all 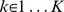 and 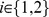. We define a feasible solution as a solution of the form 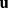 given above that satisfies, in addition, the constraint
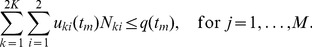
(7)


This represents the fact that at any control time 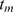, there is a finite amount of vaccine 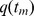 to be used that cannot be exceeded. Let 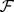 be the set of all feasible solutions, and denote by 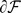 the boundary of 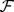.

Our aim is to minimize the expected number of people who become infected and ill, denoted by the objective function 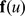. Hence, we wish to find solutions to the following optimization problem:
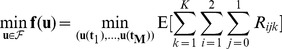
(8)subject to the constraint (7). 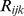 represents the number of recovered symptomatic in subgroup 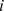 (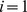 for children and 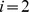 for adults) and vaccinated status 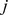 (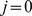 for unvaccinated and 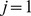 for vaccinated).

#### Genetic algorithm

We use a genetic algorithm [53], [54] to compute the optimal strategies. In our case, a chromosome represents a solution to our optimization problem, and we can think of a gene as a particular control vector. We initialize the genetic algorithm by randomly generating 48 feasible solutions (Text S1). In addition, we add two particular solutions. The first particular solution consists of a pro rata distribution of resources. Here, we distribute the available vaccine among all the cities and all age-groups, proportionally to the size of that age-group. The second particular solution consists of a children-only pro rata distribution. Here, we distribute vaccine among children only, proportionally to the number of children with respect to the total child population in the network. This procedure ensures that our optimizer has a variety of strategies to choose from, and that it takes into account the observation that prioritizing vaccination in children can lead to the optimal use of resources [12], [55], [27], [15], [56], [57].

In each generation, we use the objective function 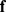 to determine the fitness of each solution. To ensure that each generation has better (more fit) individuals than its predecessor, we carry over the best 25 chromosomes from one generation directly to the next one. We then use a crossover method to create the remaining set of 25 new chromosomes. Finally, the chromosomes undergoe a mutation.

Because the new chromosome likely will not satisfy constraint (7), we incorporate an extra step in the genetic algorithm. At this stage, the chromosome is transformed just before its evaluation by mapping each gene to the boundary of its feasible region.

Define the transformation 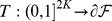 by
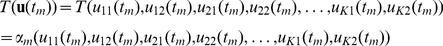
(9)where 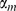 is given by
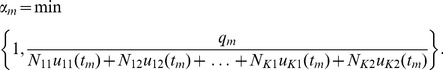
(10)


This transformation maps radially each vector to its corresponding vector on the boundary of the feasible region. The minimum is taken to ensure that no transformed gene has a coordinate greater than one, which would imply in our case vaccinating more than 100% of the population.

## Supporting Information

Figure S1 **Epidemic curves for the 16 cities considered in the baseline case.** The epidemic is started in Jakarta, with 10 infectious individuals.
(TIF)

Figure S2 **Attack rate with 95% bootstrapped CI for a single intervention for six different vaccination days considered and six different vaccination coverages for an epidemic starting in Hong Kong.** Each panel represents a given number of vaccine doses available to distribute in the entire network: A) Two million doses. B) Four million doses. C) Five million doses. D) Six million doses. E) Seven million doses. F) Ten million doses. For each panel, each point in the graph corresponds to the attack rate for a single vaccination day, either on day 5, 10, 15, 30, 60, or 90 after the beginning of the epidemic. Three different allocations are shown in each panel. The optimal strategy (blue) is the one given by our method. The pro rata strategy (green) consists of distributing vaccine to each age-group in each city proportional to the age-group population size. The children-only pro rata strategy (orange) consists of distributing vaccine only to children in each city proportional to the children's population size. The baseline scenario (red) indicates no vaccination. For early vaccination, an epidemic starting in Hong Kong yield to a higher attack rates for the optimal and children only pro rata solutions, this is due to the fact that the flux of daily travelers through Hong Kong is much higher than the flux through Jakarta.
(TIF)

Figure S3 **Epidemic prevention potential (EPP) starting in Hong Kong with 95% bootstrapped CI.** Three different allocations are shown in each panel. Each panel represents a given number of vaccine doses available to distribute in the network. A) Two million doses. B) Four million doses. C) Five million doses. D) Six million doses. E) Seven million doses. F) Ten million doses. Each point in each graph corresponds to the EPP for a single vaccination day, either on day 5, 10, 15, 30, 60, or 90 after the beginning of the epidemic. The optimal strategy (blue) is the one given by our method. The pro rata strategy (green) consists of distributing vaccine to each age-group in each city proportional to the age-group population size. The children-only pro rata strategy (orange) consists of distributing vaccine only to children in each city proportional to the children's population size. When less than 10 million doses are available, the EPP for an epidemic starting in Hong Kong is considerably lower for than if the epidemic starting in Jakarta, highlighting the fact that it is more difficult to mitigate an epidemic if it starts in a more connected city.
(TIF)

Figure S4 **Attack rate with 95% bootstrapped CI for a single intervention for six different vaccination days considered and six different vaccination coverages for an epidemic starting in Taipei.** Each panel represents a given number of vaccine doses available to distribute in the entire network: A) Two million doses. B) Four million doses. C) Five million doses. D) Six million doses. E) Seven million doses. F) Ten million doses. For each panel, each point in the graph corresponds to the attack rate for a single vaccination day, either on day 5, 10, 15, 30, 60, or 90 after the beginning of the epidemic. Three different allocations are shown in each panel. The optimal strategy (blue) is the one given by our method. The pro rata strategy (green) consists of distributing vaccine to each age-group in each city proportional to the age-group population size. The children-only pro rata strategy (orange) consists of distributing vaccine only to children in each city proportional to the children's population size. The baseline scenario (red) indicates no vaccination. The attack rates under this scenario are similar to those when the epidemic is seeded in Hong Kong.
(TIF)

Figure S5 **Epidemic prevention potential (EPP) starting in Taipei with 95% bootstrapped CI.** Three different allocations are shown in each panel. Each panel represents a given number of vaccine doses available to distribute in the network. A) Two million doses. B) Four million doses. C) Five million doses. D) Six million doses. E) Seven million doses. F) Ten million doses. Each point in each graph corresponds to the EPP for a single vaccination day, either on day 5, 10, 15, 30, 60, or 90 after the beginning of the epidemic. The optimal strategy (blue) is the one given by our method. The pro rata strategy (green) consists of distributing vaccine to each age-group in each city proportional to the age-group population size. The children-only pro rata strategy (orange) consists of distributing vaccine only to children in each city proportional to the children's population size. The EPP here is very similar to the one obtained when the epidemic starts in Hong Kong.
(TIF)

Figure S6
**Attack rate with 95% bootstrapped CI for a single intervention for 6 different vaccination days considered and 6 different vaccination coverages for an epidemic with** 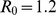 **. Here, the epidemic was seeded in Jakarta.** Each panel represents a given number of vaccine doses available to distribute in the entire network: A) Two million doses. B) Four million doses. C) Five million doses. D) Six million doses. E) Seven million doses. F) Ten million doses. For each panel, each point in the graph corresponds to the attack rate for a single vaccination day, either on day 5, 10, 15, 30, 60, or 90 after the beginning of the epidemic. Three different allocations are shown in each panel: The optimal strategy (blue) is the one given by our method. The pro rata strategy (green) consists of distributing vaccine to each age-group in each city proportional to the age-group population size. The children-only pro rata strategy (orange) consists of distributing vaccine only to children in each city proportional to the children's population size. The baseline scenario (red) indicates no vaccination. As expected, a low 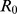 requires few doses of vaccine to mitigate an epidemic: with four million of doses of vaccine, all strategies considered yield an attack rate of less than 2% of the total population.(TIF)

Figure S7
**Epidemic prevention potential (EPP) for** 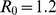 **with 95% bootstrapped CI**. Here, the epidemic was seeded in Jakarta. Each panel represents a given number of vaccine doses available to distribute in the network. A) Two million doses. B) Four million doses. C) Five million doses. D) Six million doses. E) Seven million doses. F) Ten million doses. Each point in each graph corresponds to the EPP for a single vaccination day, either on day 5, 10, 15, 30, 60, or 90 after the beginning of the epidemic. Three different strategies are shown in each panel. The optimal strategy (blue) is the one given by our method. The pro rata strategy (green) consists of distributing vaccine to each age-group in each city proportional to the age-group population size. The children-only pro rata strategy (orange) consists of distributing vaccine only to children in each city proportional to the children's population size. The optimal and the children-only pro rata strategy can mitigate most of the epidemics with as few as four million doses.(TIF)

Figure S8
**Attack rate with 95% bootstrapped CI for a single intervention for 6 different vaccination days considered and 6 different vaccination coverages for an epidemic with** 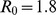 **.** Here, the epidemic was seeded in Jakarta. Each panel represents a given number of vaccine doses available to distribute in the entire network: A) Two million doses. B) Four million doses. C) Five million doses. D) Six million doses. E) Seven million doses. F) Ten million doses. For each panel, each point in the graph corresponds to the attack rate for a single vaccination day, either on day 5, 10, 15, 30, 60, or 90 after the beginning of the epidemic. Three different allocations are shown in each panel: The optimal strategy (blue) is the one given by our method. The pro rata strategy (green) consists of distributing vaccine to each age-group in each city proportional to the age-group population size. The children-only pro rata strategy (orange) consists of distributing vaccine only to children in each city proportional to the children's population size. The baseline scenario (red) indicates no vaccination.(TIF)

Figure S9
**Epidemic prevention potential (EPP) for** 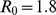 **with 95% bootstrapped CI and the epidemic was seeded in Jakarta**. Each panel represents a given number of vaccine doses available to distribute in the network. A) Two million doses. B) Four million doses. C) Five million doses. D) Six million doses. E) Seven million doses. F) Ten million doses. Each point in each graph corresponds to the EPP for a single vaccination day, either on day 5, 10, 15, 30, 60, or 90 after the beginning of the epidemic. Three different allocations are shown in each panel: The optimal strategy (blue) is the one given by our method. The pro rata strategy (green) consists of distributing vaccine to each age-group in each city proportional to the age-group population size. The children-only pro rata strategy (orange) consists of distributing vaccine only to children in each city proportional to the children's population size. Here, the EPP is much lower than in the base case scenarios (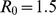). The optimal strategy is the only strategy able to mitigate some of the epidemics for all days and coverages considered.(TIF)

Figure S10 **Attack rate with 95% bootstrapped CI for five million doses with different travel probabilities.** A) An infectious symptomatic individual is 10% less likely to travel than an asymptomatic individual. B) An infectious symptomatic individual is 75% less likely to travel than an asymptomatic individual. For each panel, each point in the graph corresponds to the attack rate for a single vaccination day, either on day 5, 10, 15, 30, 60, or 90 after the beginning of the epidemic.
(TIF)

Figure S11 **Epidemic prevention potential (EPP) with 95% bootstrapped CI for five million doses with different travel probabilities.** A) An infectious symptomatic individual is 10% less likely to travel than an asymptomatic individual. B) An infectious symptomatic individual is 75% less likely to travel than an asymptomatic individual. For each panel, each point in the graph corresponds to the attack rate for a single vaccination day, either on day 5, 10, 15, 30, 60, or 90 after the beginning of the epidemic.
(TIF)

Figure S12 **Results for five million doses when children have a 50% reduction in their probability of travel.** A) Attack rates with 95% bootstrapped CI. B) EPP with 95% bootstrapped CI. For each panel, each point in the graph corresponds to the attack rate for a single vaccination day, either on day 5, 10, 15, 30, 60, or 90 after the beginning of the epidemic.
(TIF)

Figure S13
**Attack rate with 95% bootstrapped CI for five million doses with lower vaccine efficacies.** A) One-third of their original values (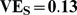, 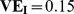 and 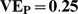). B) Two-thirds of their original values (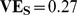, 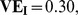 and 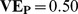). For each panel, each point in the graph corresponds to the attack rate for a single vaccination day, either on day 5, 10, 15, 30, 60, or 90 after the beginning of the epidemic.(TIF)

Figure S14
**Epidemic prevention potential (EPP) with 95% bootstrapped CI for five million doses with lower vaccine efficacies.** A) One-third of their original values (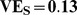, 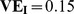 and 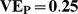). B) Two-thirds of their original values (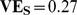, 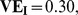 and 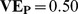). For each panel, each point in the graph corresponds to the attack rate for a single vaccination day, either on day 5, 10, 15, 30, 60, or 90 after the beginning of the epidemic.(TIF)

Figure S15 **Results for five million doses when vaccination is completed in 10 days.** A) Attack rates with 95% bootstrapped CI. B) EPP with 95% bootstrapped CI. For each panel, each point in the graph corresponds to the attack rate for a single vaccination day, either on day 5, 10, 15, 30, 60, or 90 after the beginning of the epidemic.
(TIF)

Figure S16 **Results for five million doses when vaccines are given to children only.** A) Attack rates with 95% bootstrapped CI. B) EPP with 95% bootstrapped CI. For each panel, each point in the graph corresponds to the attack rate for a single vaccination day, either on day 5, 10, 15, 30, 60, or 90 after the beginning of the epidemic.
(TIF)

Text S1 **Complete mathematical model and sensitivity analysis.**
(PDF)
