# Supplementary figures and images for: Optimal Vaccine Allocation for the Early Mitigation of Pandemic Influenza
Source: PLoS Comput Biol. 2013 Mar 21;9(3):e1002964. doi: 10.1371/journal.pcbi.1002964 (PMC3605056; doi:10.1371/journal.pcbi.1002964)

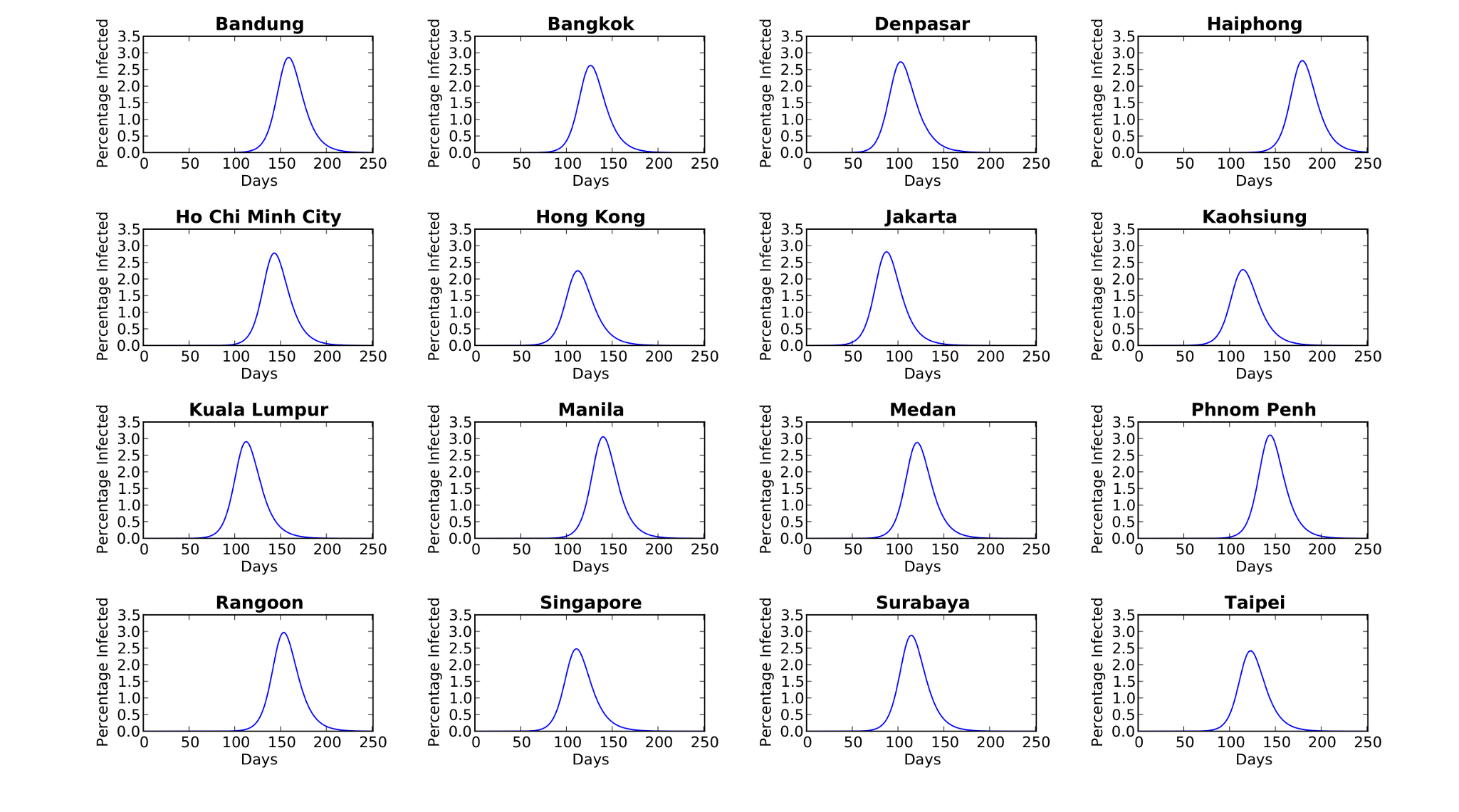

Supplement: Figure S1 — Epidemic curves for the 16 cities considered in the baseline case. The epidemic is started in Jakarta, with 10 infectious individuals. (TIF) [file pcbi.1002964.s001.tif]

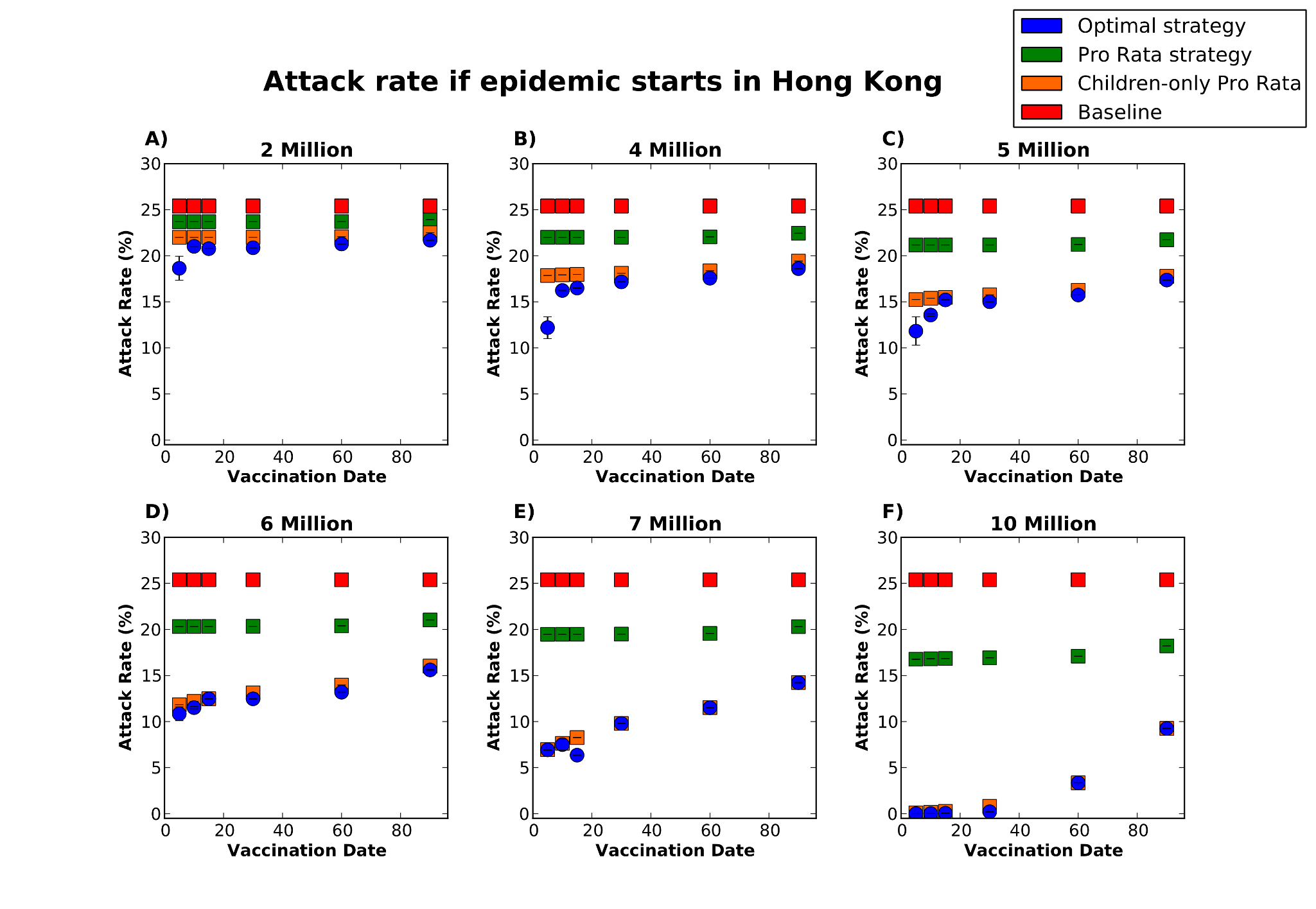

Supplement: Figure S2 — Attack rate with 95% bootstrapped CI for a single intervention for six different vaccination days considered and six different vaccination coverages for an epidemic starting in Hong Kong. Each panel represents a given number of vaccine doses available to distribute in the entire network: A) Two million doses. B) Four million doses. C) Five million doses. D) Six million doses. E) Seven million doses. F) Ten million doses. For each panel, each point in the graph corresponds to the attack rate for a single vaccination day, either on day 5, 10, 15, 30, 60, or 90 after the beginning of the epidemic. Three different allocations are shown in each panel. The optimal strategy (blue) is the one given by our method. The pro rata strategy (green) consists of distributing vaccine to each age-group in each city proportional to the age-group population size. The children-only pro rata strategy (orange) consists of distributing vaccine only to children in each city proportional to the children's population size. The baseline scenario (red) indicates no vaccination. For early vaccination, an epidemic starting in Hong Kong yield to a higher attack rates for the optimal and children only pro rata solutions, this is due to the fact that the flux of daily travelers through Hong Kong is much higher than the flux through Jakarta. (TIF) [file pcbi.1002964.s002.tif]

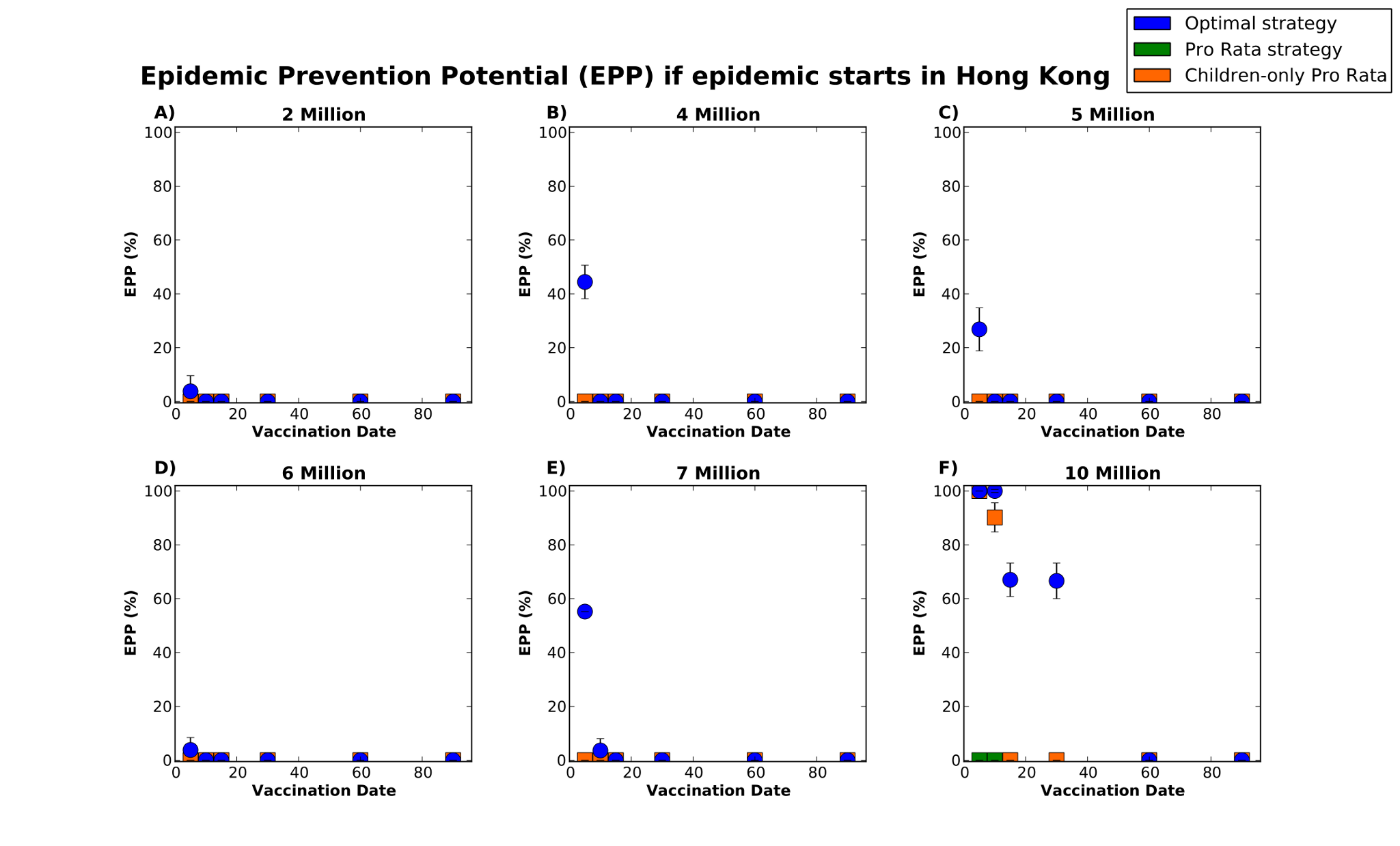

Supplement: Figure S3 — Epidemic prevention potential (EPP) starting in Hong Kong with 95% bootstrapped CI. Three different allocations are shown in each panel. Each panel represents a given number of vaccine doses available to distribute in the network. A) Two million doses. B) Four million doses. C) Five million doses. D) Six million doses. E) Seven million doses. F) Ten million doses. Each point in each graph corresponds to the EPP for a single vaccination day, either on day 5, 10, 15, 30, 60, or 90 after the beginning of the epidemic. The optimal strategy (blue) is the one given by our method. The pro rata strategy (green) consists of distributing vaccine to each age-group in each city proportional to the age-group population size. The children-only pro rata strategy (orange) consists of distributing vaccine only to children in each city proportional to the children's population size. When less than 10 million doses are available, the EPP for an epidemic starting in Hong Kong is considerably lower for than if the epidemic starting in Jakarta, highlighting the fact that it is more difficult to mitigate an epidemic if it starts in a more connected city. (TIF) [file pcbi.1002964.s003.tif]

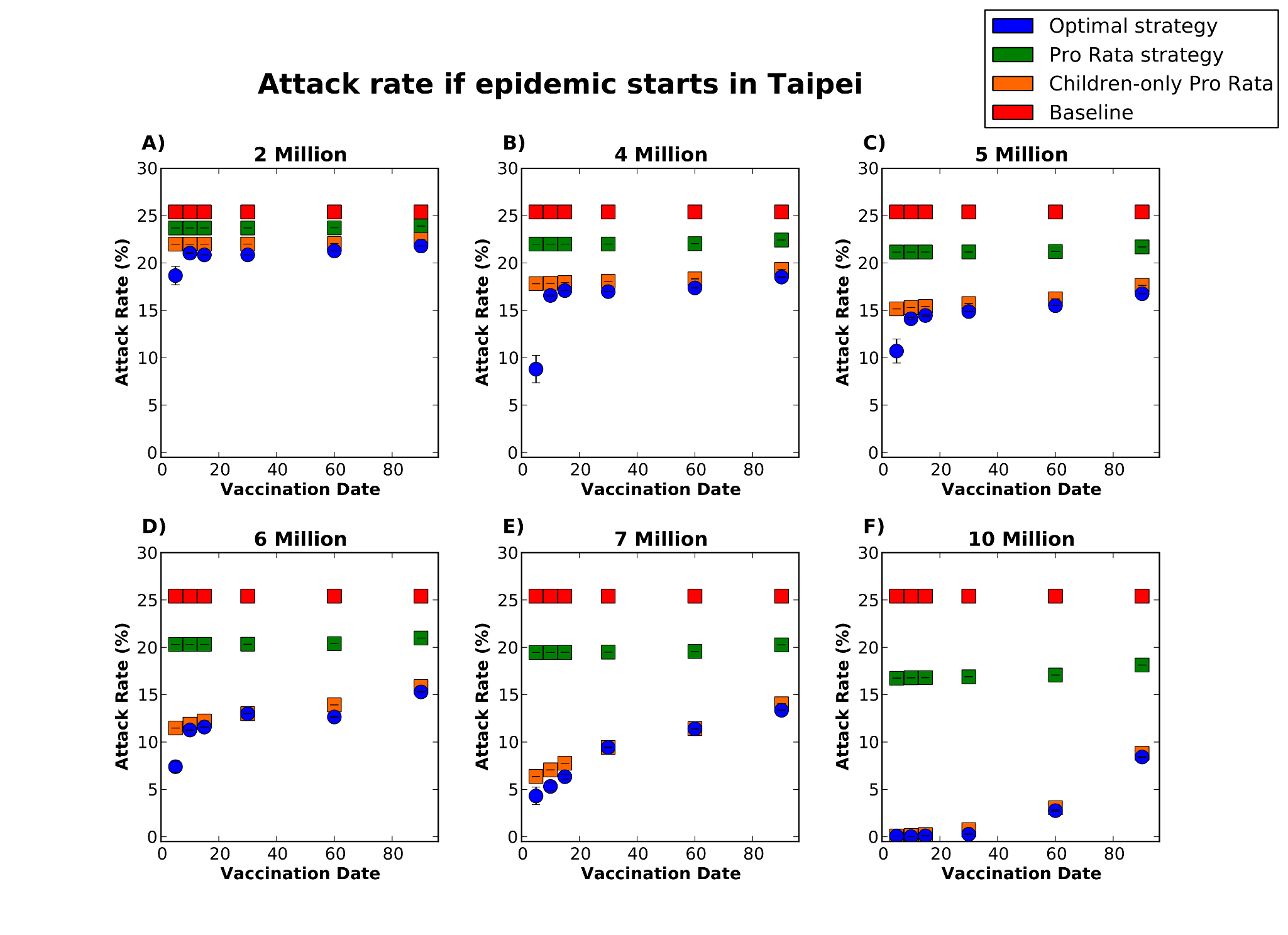

Supplement: Figure S4 — Attack rate with 95% bootstrapped CI for a single intervention for six different vaccination days considered and six different vaccination coverages for an epidemic starting in Taipei. Each panel represents a given number of vaccine doses available to distribute in the entire network: A) Two million doses. B) Four million doses. C) Five million doses. D) Six million doses. E) Seven million doses. F) Ten million doses. For each panel, each point in the graph corresponds to the attack rate for a single vaccination day, either on day 5, 10, 15, 30, 60, or 90 after the beginning of the epidemic. Three different allocations are shown in each panel. The optimal strategy (blue) is the one given by our method. The pro rata strategy (green) consists of distributing vaccine to each age-group in each city proportional to the age-group population size. The children-only pro rata strategy (orange) consists of distributing vaccine only to children in each city proportional to the children's population size. The baseline scenario (red) indicates no vaccination. The attack rates under this scenario are similar to those when the epidemic is seeded in Hong Kong. (TIF) [file pcbi.1002964.s004.tif]

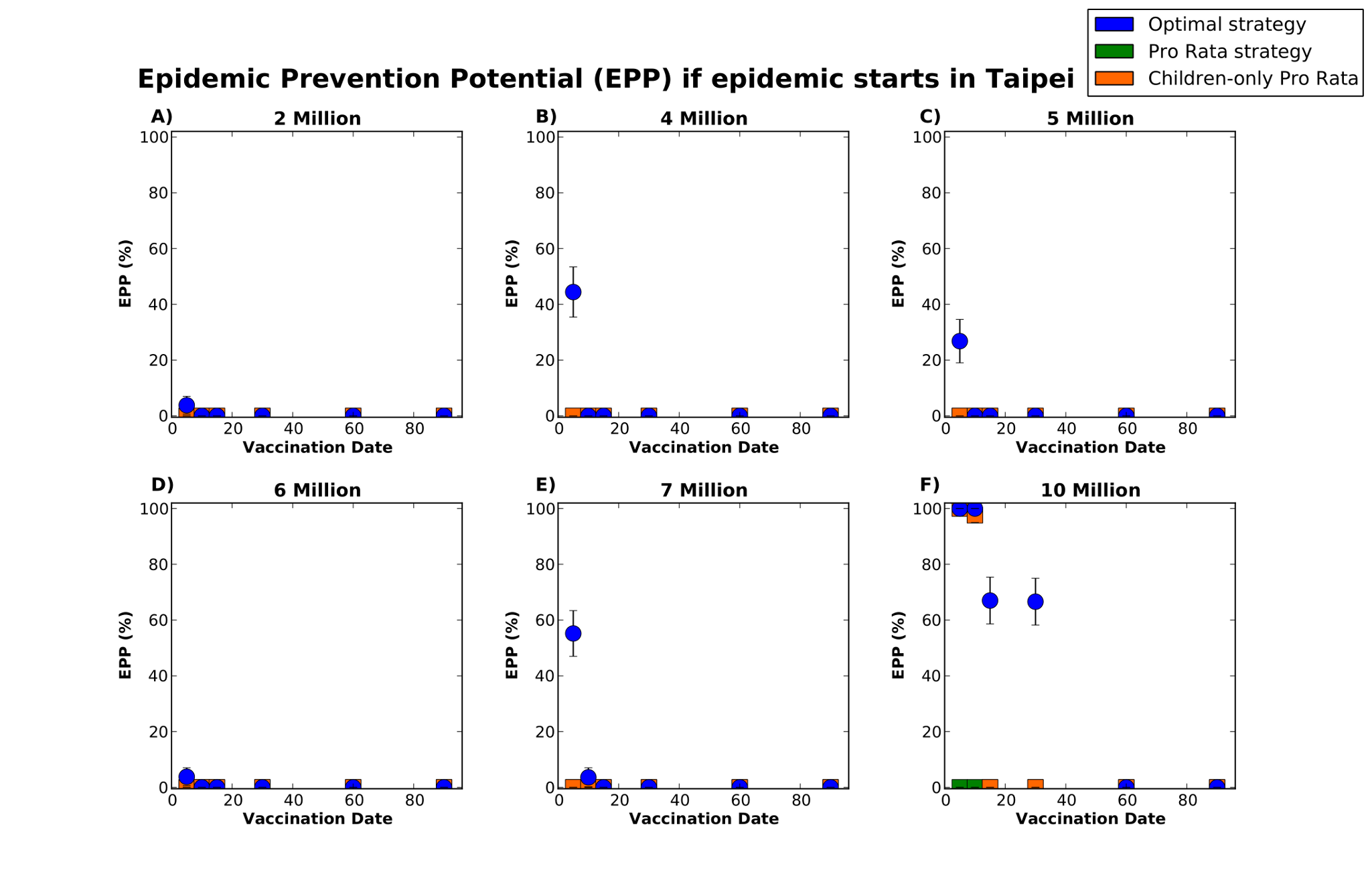

Supplement: Figure S5 — Epidemic prevention potential (EPP) starting in Taipei with 95% bootstrapped CI. Three different allocations are shown in each panel. Each panel represents a given number of vaccine doses available to distribute in the network. A) Two million doses. B) Four million doses. C) Five million doses. D) Six million doses. E) Seven million doses. F) Ten million doses. Each point in each graph corresponds to the EPP for a single vaccination day, either on day 5, 10, 15, 30, 60, or 90 after the beginning of the epidemic. The optimal strategy (blue) is the one given by our method. The pro rata strategy (green) consists of distributing vaccine to each age-group in each city proportional to the age-group population size. The children-only pro rata strategy (orange) consists of distributing vaccine only to children in each city proportional to the children's population size. The EPP here is very similar to the one obtained when the epidemic starts in Hong Kong. (TIF) [file pcbi.1002964.s005.tif]

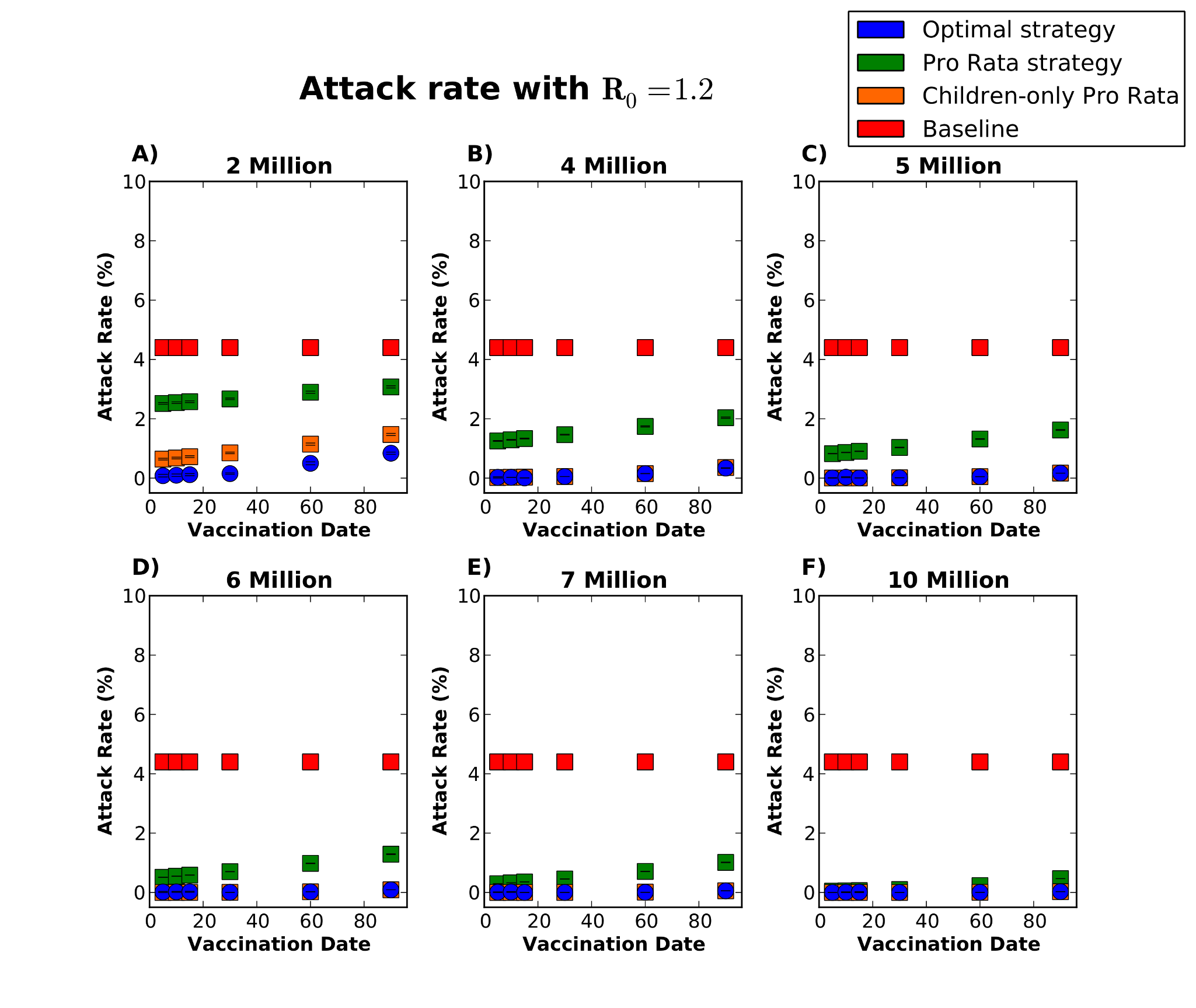

Supplement: Figure S6 — Attack rate with 95% bootstrapped CI for a single intervention for 6 different vaccination days considered and 6 different vaccination coverages for an epidemic with . Here, the epidemic was seeded in Jakarta. Each panel represents a given number of vaccine doses available to distribute in the entire network: A) Two million doses. B) Four million doses. C) Five million doses. D) Six million doses. E) Seven million doses. F) Ten million doses. For each panel, each point in the graph corresponds to the attack rate for a single vaccination day, either on day 5, 10, 15, 30, 60, or 90 after the beginning of the epidemic. Three different allocations are shown in each panel: The optimal strategy (blue) is the one given by our method. The pro rata strategy (green) consists of distributing vaccine to each age-group in each city proportional to the age-group population size. The children-only pro rata strategy (orange) consists of distributing vaccine only to children in each city proportional to the children's population size. The baseline scenario (red) indicates no vaccination. As expected, a low requires few doses of vaccine to mitigate an epidemic: with four million of doses of vaccine, all strategies considered yield an attack rate of less than 2% of the total population. (TIF) [file pcbi.1002964.s006.tif]

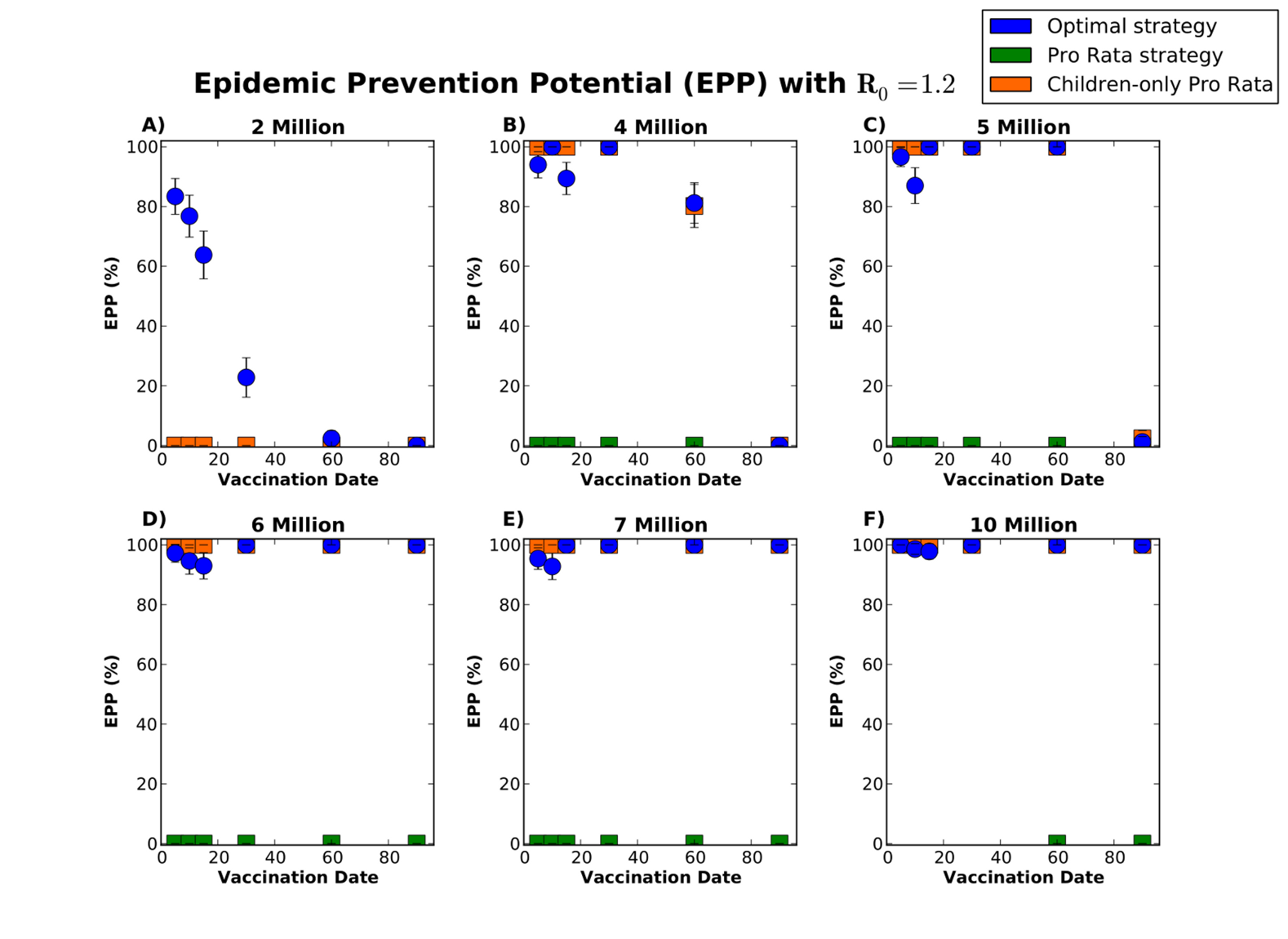

Supplement: Figure S7 — Epidemic prevention potential (EPP) for with 95% bootstrapped CI. Here, the epidemic was seeded in Jakarta. Each panel represents a given number of vaccine doses available to distribute in the network. A) Two million doses. B) Four million doses. C) Five million doses. D) Six million doses. E) Seven million doses. F) Ten million doses. Each point in each graph corresponds to the EPP for a single vaccination day, either on day 5, 10, 15, 30, 60, or 90 after the beginning of the epidemic. Three different strategies are shown in each panel. The optimal strategy (blue) is the one given by our method. The pro rata strategy (green) consists of distributing vaccine to each age-group in each city proportional to the age-group population size. The children-only pro rata strategy (orange) consists of distributing vaccine only to children in each city proportional to the children's population size. The optimal and the children-only pro rata strategy can mitigate most of the epidemics with as few as four million doses. (TIF) [file pcbi.1002964.s007.tif]

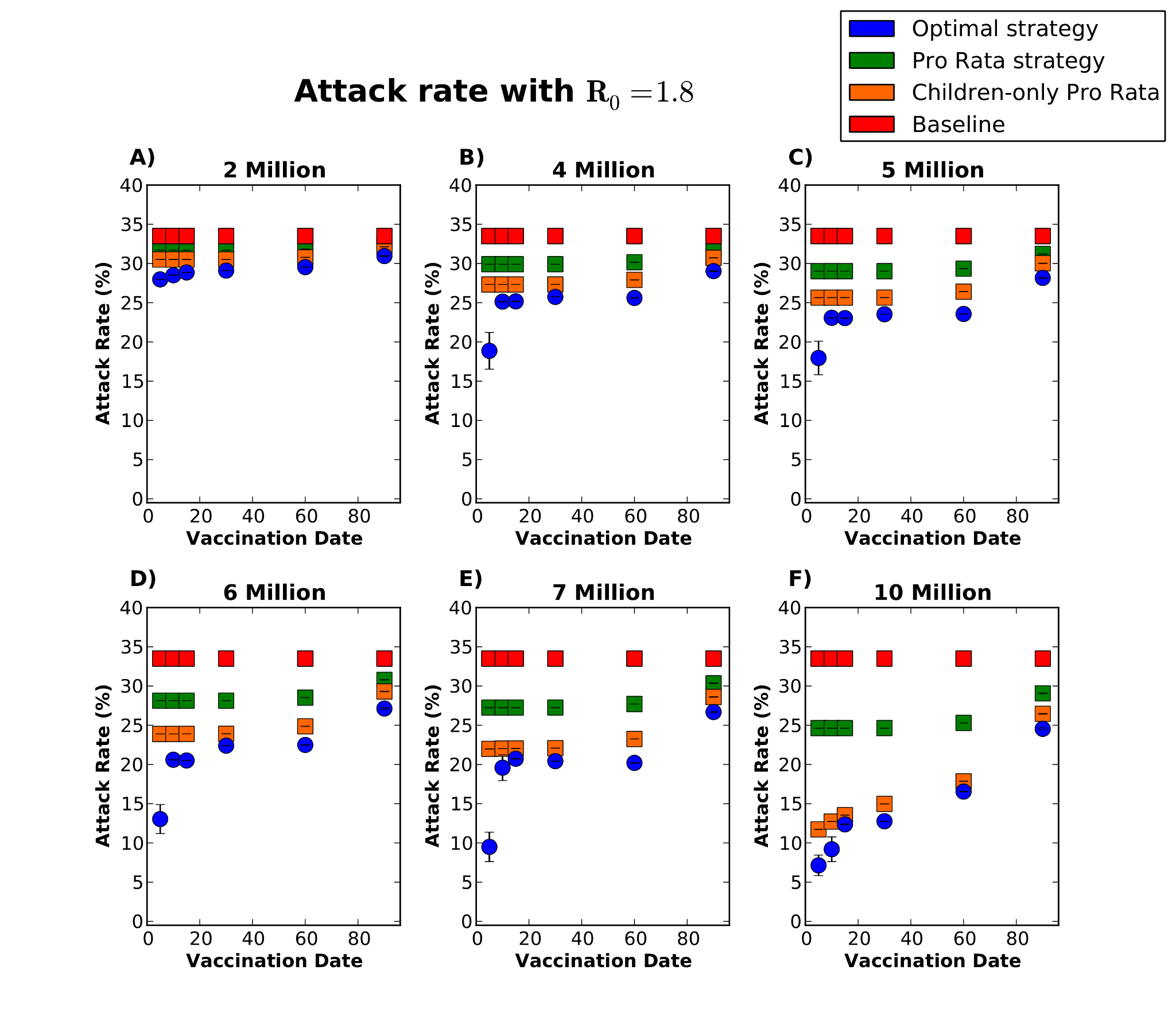

Supplement: Figure S8 — Attack rate with 95% bootstrapped CI for a single intervention for 6 different vaccination days considered and 6 different vaccination coverages for an epidemic with . Here, the epidemic was seeded in Jakarta. Each panel represents a given number of vaccine doses available to distribute in the entire network: A) Two million doses. B) Four million doses. C) Five million doses. D) Six million doses. E) Seven million doses. F) Ten million doses. For each panel, each point in the graph corresponds to the attack rate for a single vaccination day, either on day 5, 10, 15, 30, 60, or 90 after the beginning of the epidemic. Three different allocations are shown in each panel: The optimal strategy (blue) is the one given by our method. The pro rata strategy (green) consists of distributing vaccine to each age-group in each city proportional to the age-group population size. The children-only pro rata strategy (orange) consists of distributing vaccine only to children in each city proportional to the children's population size. The baseline scenario (red) indicates no vaccination. (TIF) [file pcbi.1002964.s008.tif]

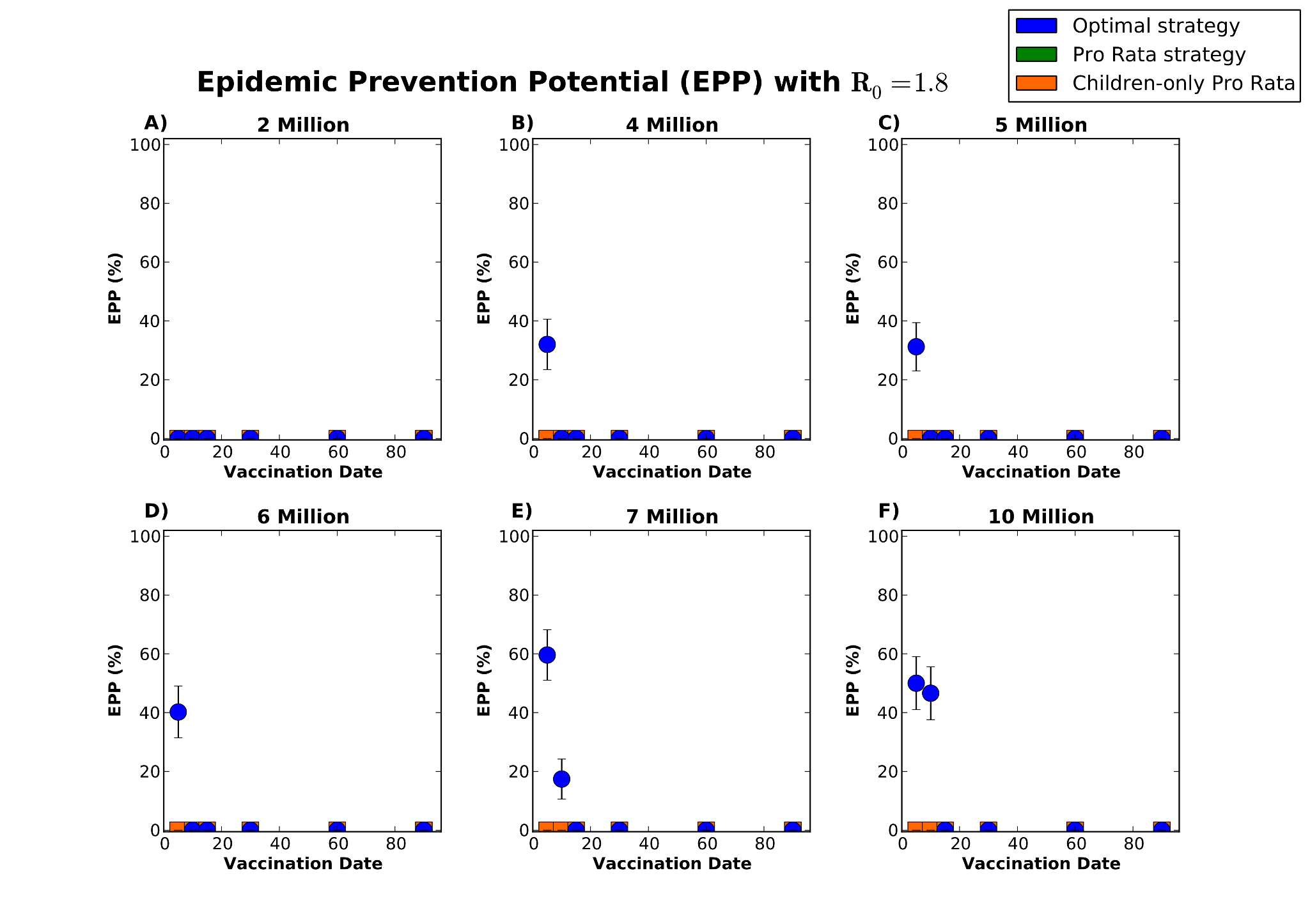

Supplement: Figure S9 — Epidemic prevention potential (EPP) for with 95% bootstrapped CI and the epidemic was seeded in Jakarta. Each panel represents a given number of vaccine doses available to distribute in the network. A) Two million doses. B) Four million doses. C) Five million doses. D) Six million doses. E) Seven million doses. F) Ten million doses. Each point in each graph corresponds to the EPP for a single vaccination day, either on day 5, 10, 15, 30, 60, or 90 after the beginning of the epidemic. Three different allocations are shown in each panel: The optimal strategy (blue) is the one given by our method. The pro rata strategy (green) consists of distributing vaccine to each age-group in each city proportional to the age-group population size. The children-only pro rata strategy (orange) consists of distributing vaccine only to children in each city proportional to the children's population size. Here, the EPP is much lower than in the base case scenarios (). The optimal strategy is the only strategy able to mitigate some of the epidemics for all days and coverages considered. (TIF) [file pcbi.1002964.s009.tif]

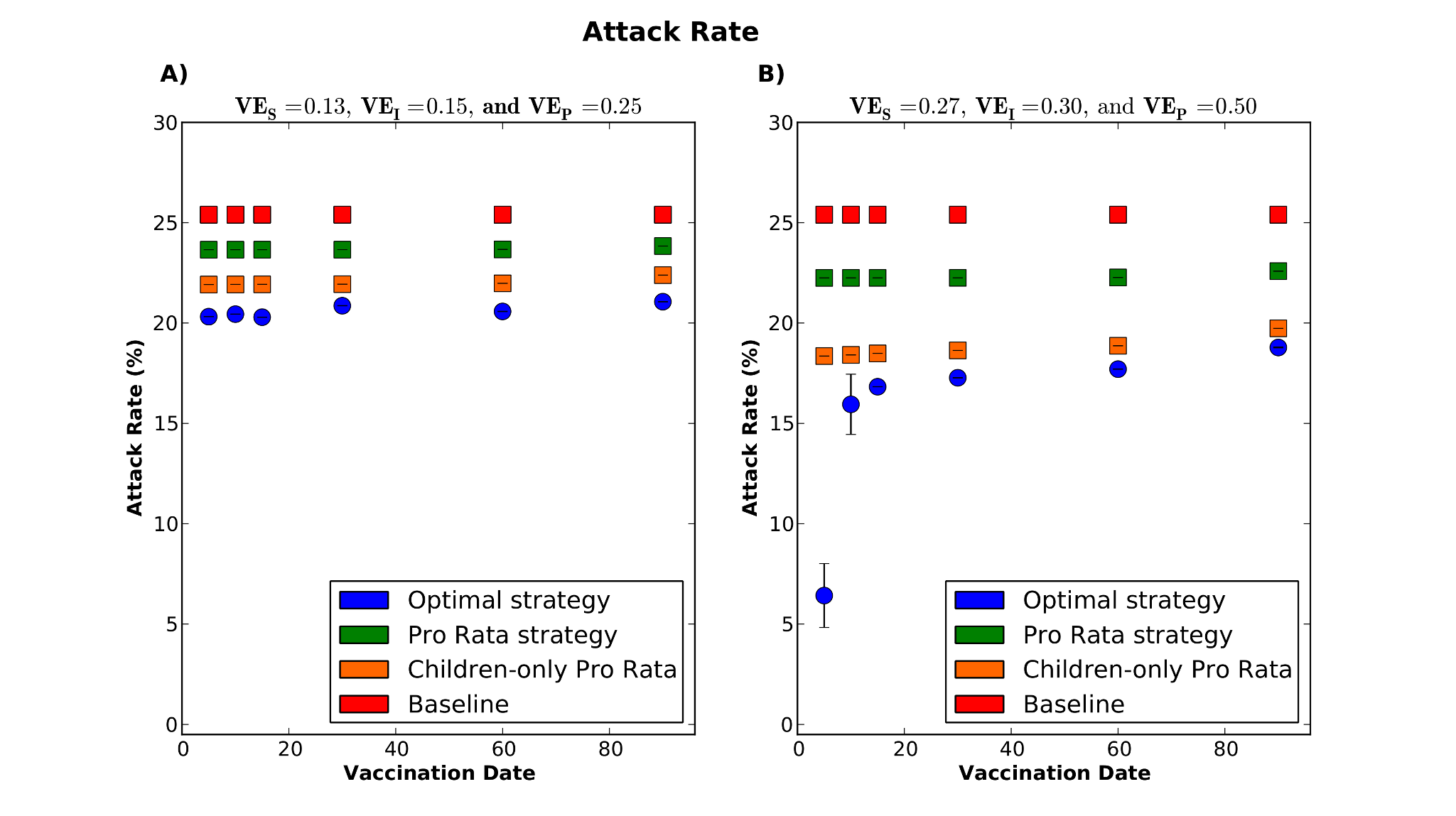

Supplement: Figure S10 — Attack rate with 95% bootstrapped CI for five million doses with different travel probabilities. A) An infectious symptomatic individual is 10% less likely to travel than an asymptomatic individual. B) An infectious symptomatic individual is 75% less likely to travel than an asymptomatic individual. For each panel, each point in the graph corresponds to the attack rate for a single vaccination day, either on day 5, 10, 15, 30, 60, or 90 after the beginning of the epidemic. (TIF) [file pcbi.1002964.s010.tif]

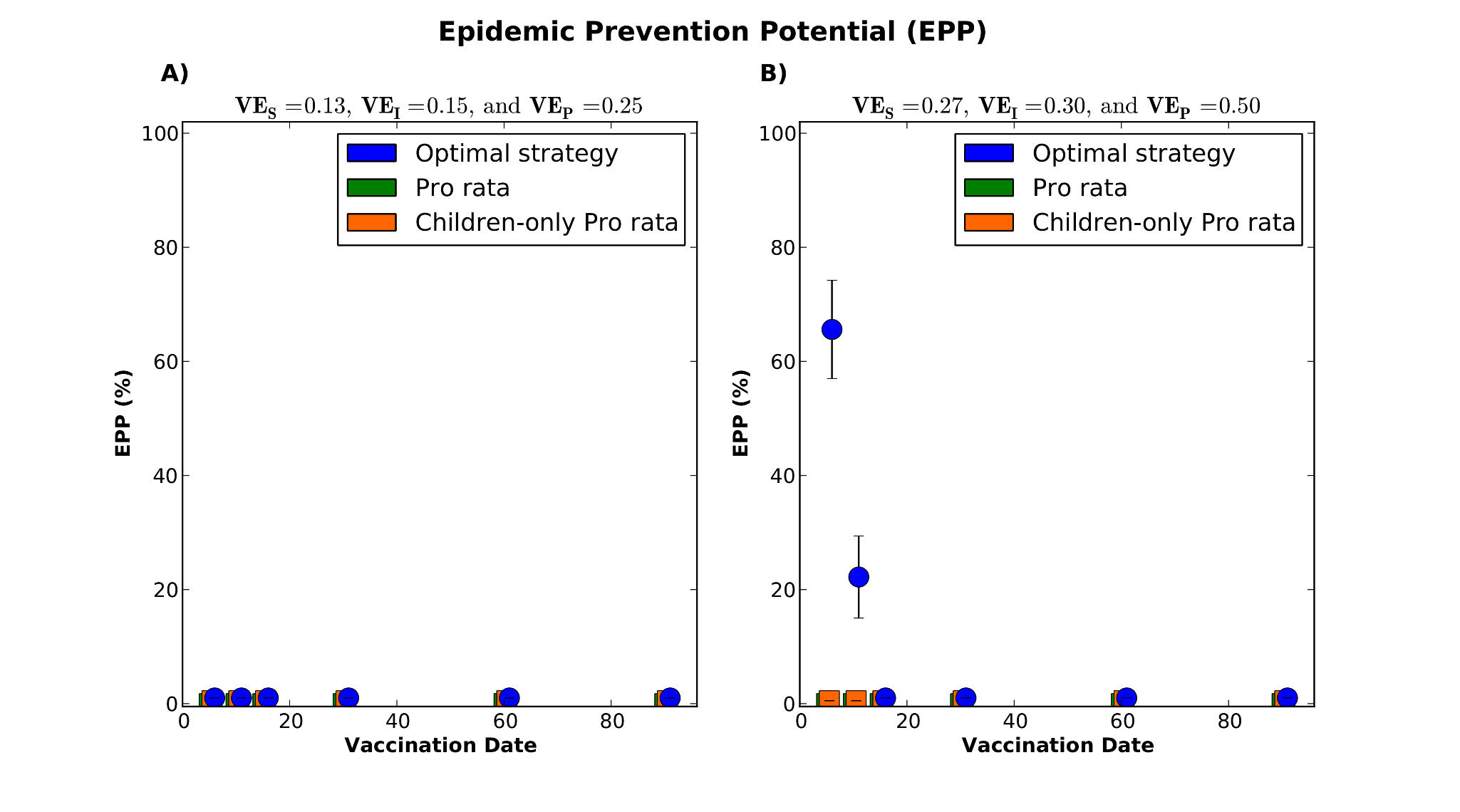

Supplement: Figure S11 — Epidemic prevention potential (EPP) with 95% bootstrapped CI for five million doses with different travel probabilities. A) An infectious symptomatic individual is 10% less likely to travel than an asymptomatic individual. B) An infectious symptomatic individual is 75% less likely to travel than an asymptomatic individual. For each panel, each point in the graph corresponds to the attack rate for a single vaccination day, either on day 5, 10, 15, 30, 60, or 90 after the beginning of the epidemic. (TIF) [file pcbi.1002964.s011.tif]

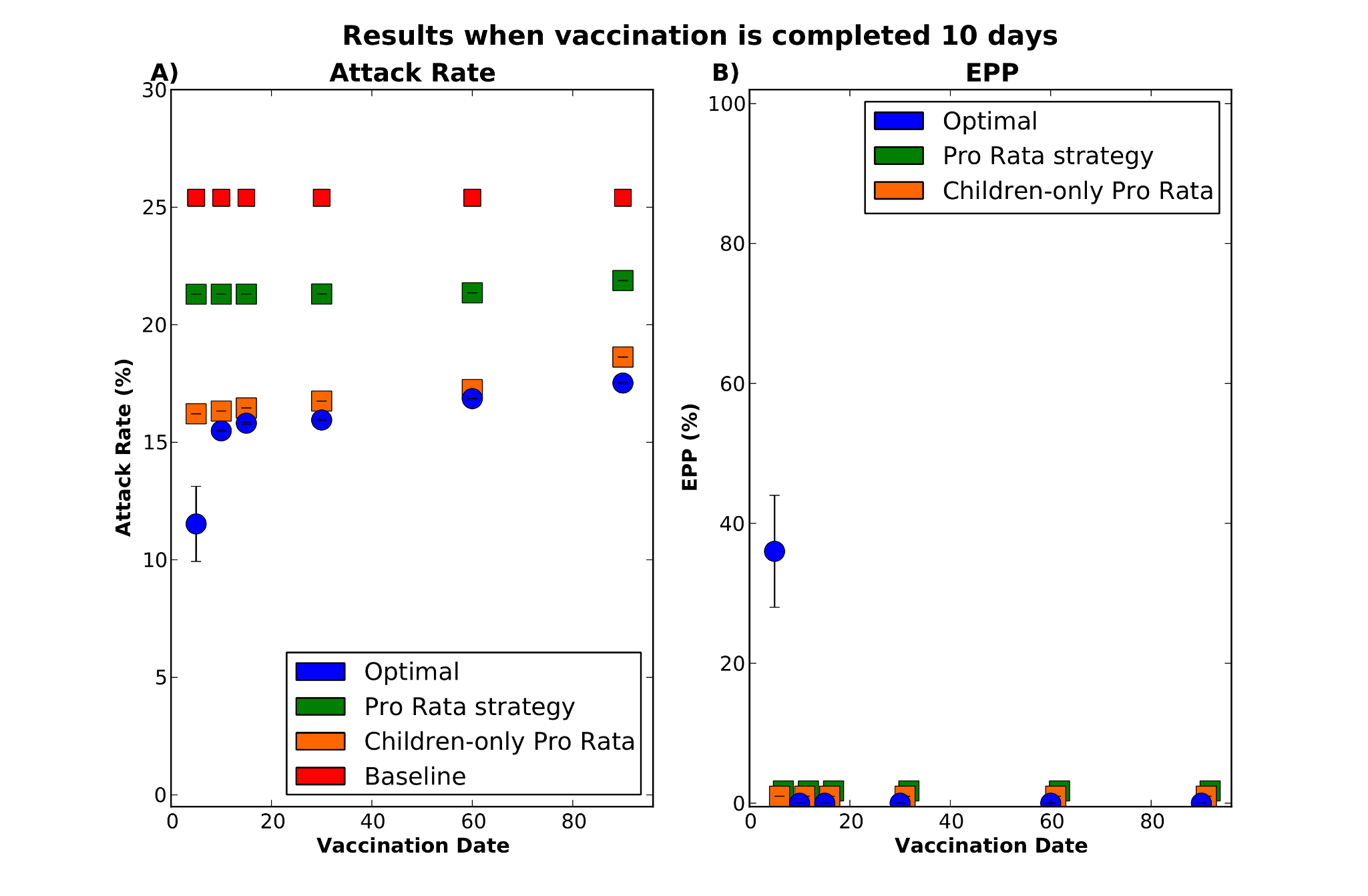

Supplement: Figure S12 — Results for five million doses when children have a 50% reduction in their probability of travel. A) Attack rates with 95% bootstrapped CI. B) EPP with 95% bootstrapped CI. For each panel, each point in the graph corresponds to the attack rate for a single vaccination day, either on day 5, 10, 15, 30, 60, or 90 after the beginning of the epidemic. (TIF) [file pcbi.1002964.s012.tif]

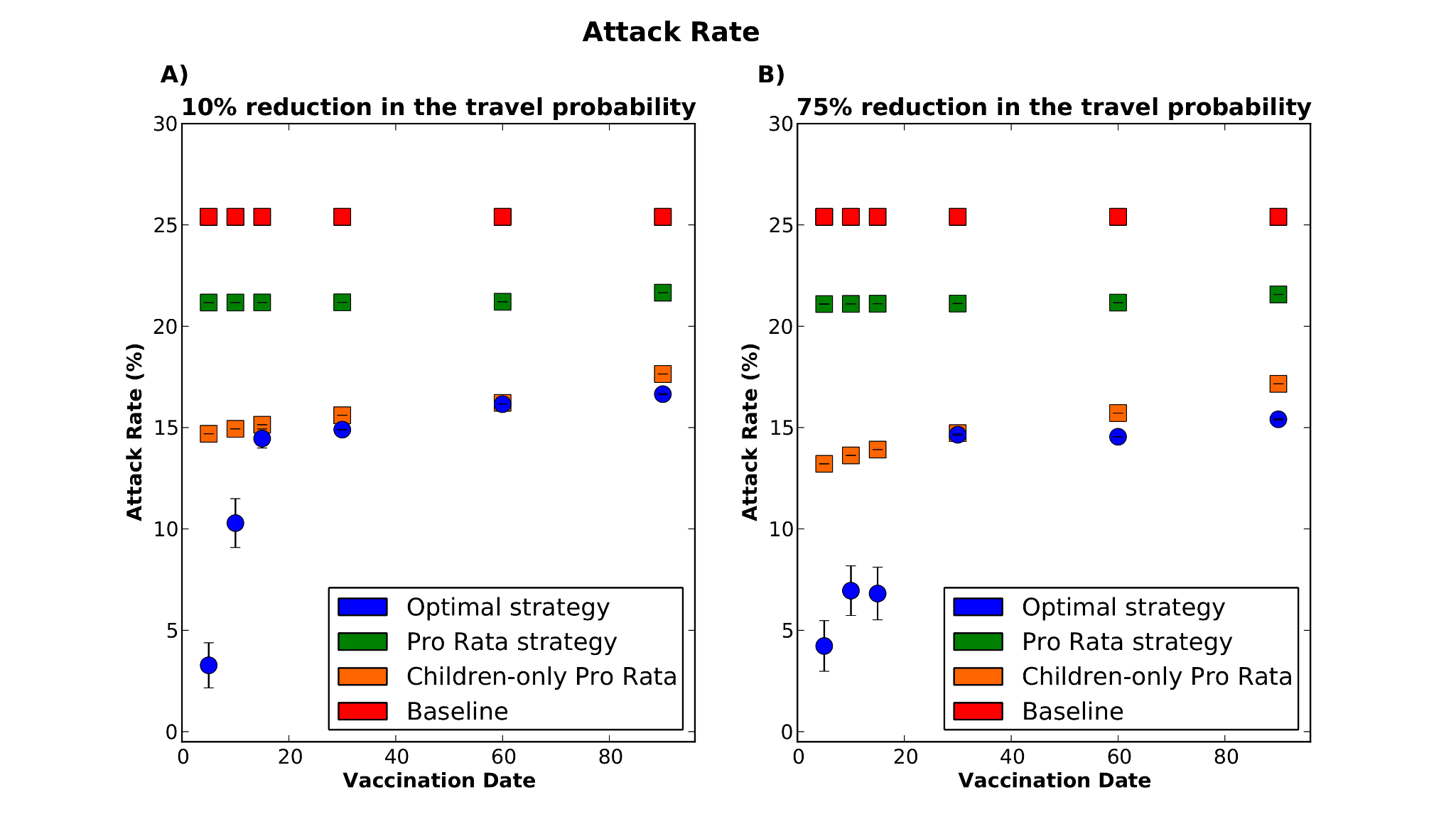

Supplement: Figure S13 — Attack rate with 95% bootstrapped CI for five million doses with lower vaccine efficacies. A) One-third of their original values (, and ). B) Two-thirds of their original values (, and ). For each panel, each point in the graph corresponds to the attack rate for a single vaccination day, either on day 5, 10, 15, 30, 60, or 90 after the beginning of the epidemic. (TIF) [file pcbi.1002964.s013.tif]

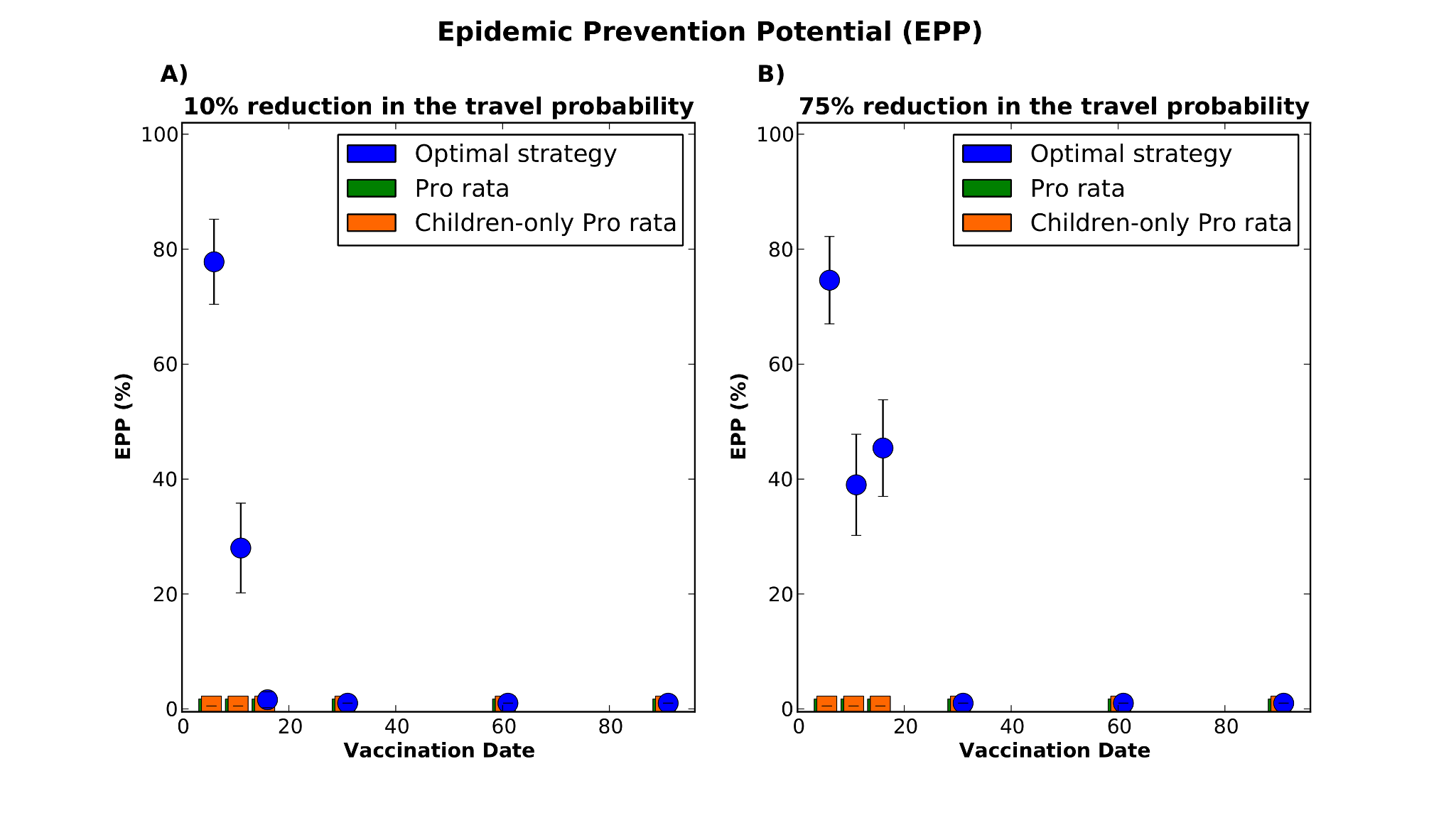

Supplement: Figure S14 — Epidemic prevention potential (EPP) with 95% bootstrapped CI for five million doses with lower vaccine efficacies. A) One-third of their original values (, and ). B) Two-thirds of their original values (, and ). For each panel, each point in the graph corresponds to the attack rate for a single vaccination day, either on day 5, 10, 15, 30, 60, or 90 after the beginning of the epidemic. (TIF) [file pcbi.1002964.s014.tif]

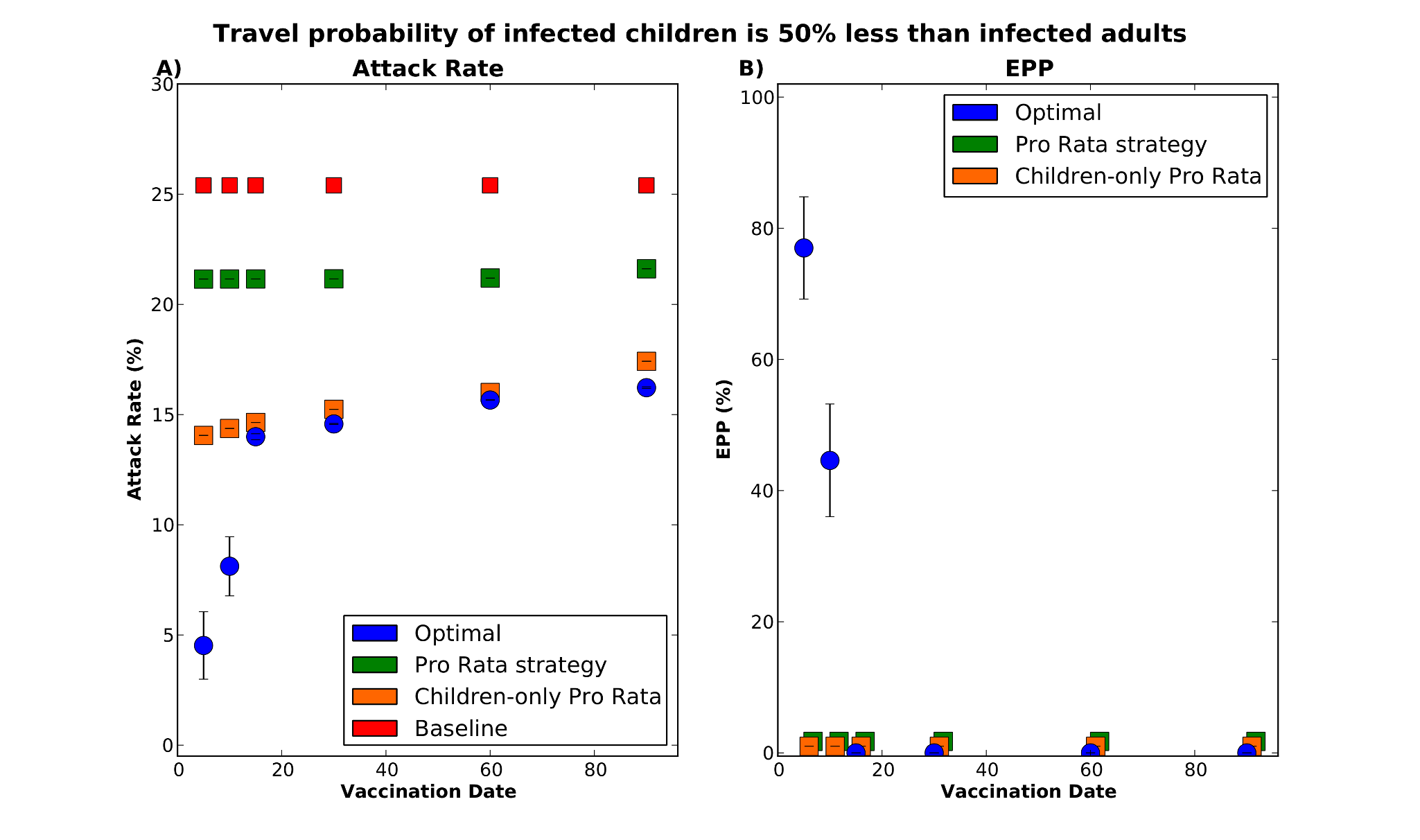

Supplement: Figure S15 — Results for five million doses when vaccination is completed in 10 days. A) Attack rates with 95% bootstrapped CI. B) EPP with 95% bootstrapped CI. For each panel, each point in the graph corresponds to the attack rate for a single vaccination day, either on day 5, 10, 15, 30, 60, or 90 after the beginning of the epidemic. (TIF) [file pcbi.1002964.s015.tif]

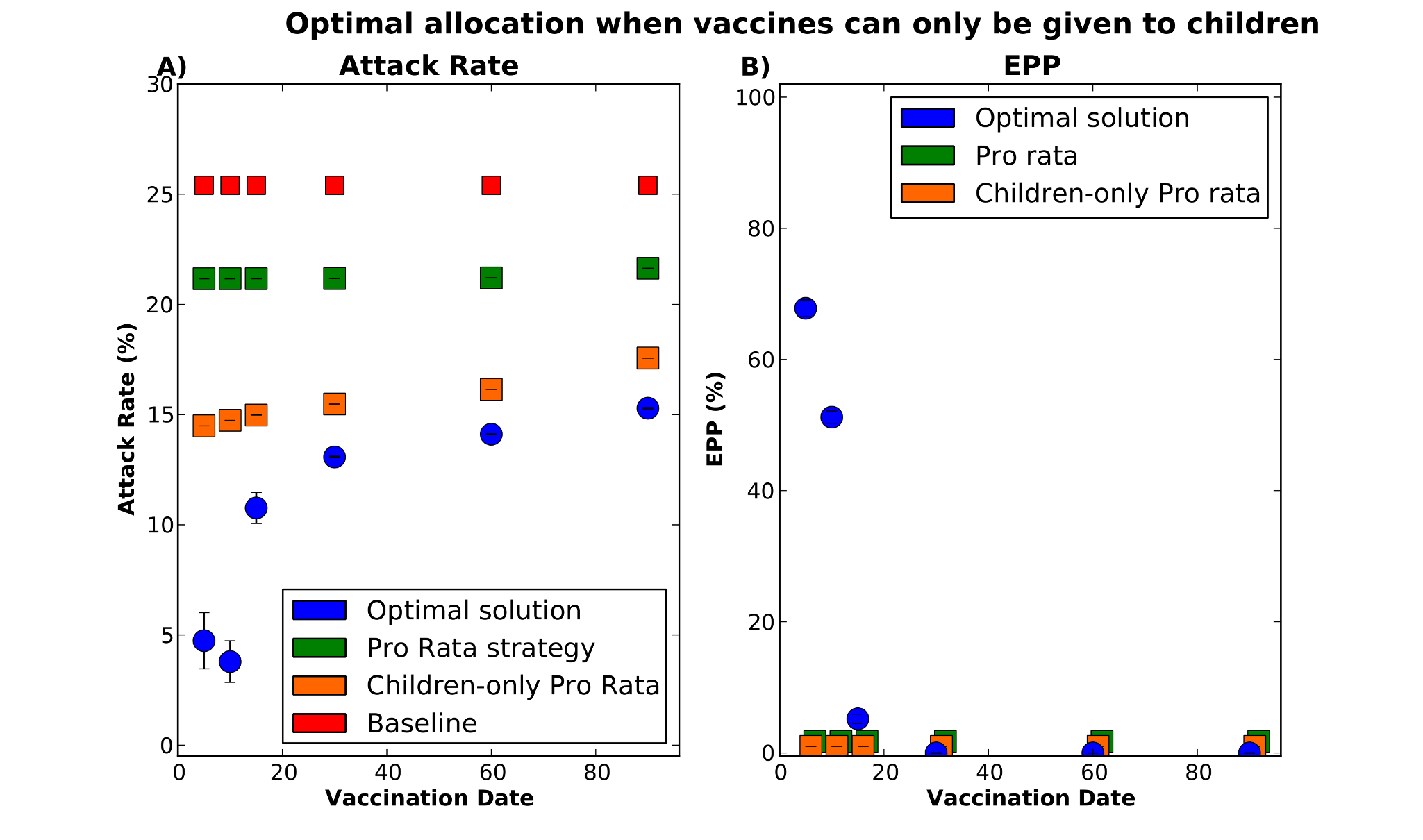

Supplement: Figure S16 — Results for five million doses when vaccines are given to children only. A) Attack rates with 95% bootstrapped CI. B) EPP with 95% bootstrapped CI. For each panel, each point in the graph corresponds to the attack rate for a single vaccination day, either on day 5, 10, 15, 30, 60, or 90 after the beginning of the epidemic. (TIF) [file pcbi.1002964.s016.tif]
