# Supplementary material for: Optimal Vaccine Allocation for the Early Mitigation of Pandemic Influenza
Source: PLoS Comput Biol. 2013 Mar 21;9(3):e1002964. doi: 10.1371/journal.pcbi.1002964 (PMC3605056; doi:10.1371/journal.pcbi.1002964)
Supplement: Text S1 — Complete mathematical model and sensitivity analysis. (PDF) [file pcbi.1002964.s017.pdf]

Supplemental Material for "Optimal vaccine allocation for the early mitigation of pandemic influenza"

Laura Matrajt[1], M. Elizabeth Halloran[2,3] Ira M. Longini Jr.[4,5*]
1 Department of Applied Mathematics, University of Washington, Seattle, WA, USA. Current address: Department of Medicine, University of Washington, Seattle, WA, 98105, USA
2 Center for Statistics and Quantitative Infectious Diseases, Vaccine and Infectious Disease Division, Fred Hutchinson Cancer Research Center, Seattle, WA, 98109, USA
3 Department of Biostatistics, School of Public Health, University of Washington, Seattle, WA, 98105, USA.
4 Emerging Pathogens Institute, University of Florida, Gainesville, FL, 32611, USA.
5 Department of Biostatistics, Colleges of Medicine, and Public Health and Health Professions, University of Florida, Gainesville, FL, 32611, USA.
$*$ E-mail: ilongini@ufl.edu

Mathematical Model

Transmission within cities

Once an epidemic has started in a city, it follows the dynamics given by a deterministic compartmental model developed in [1]. Specifically, the population in each city is divided into two age-groups, children and adults. Let N_{ki} represent the total number of children ($i = 1$) or adults ($i = 2$) in city k, and let N_k be the total population in that city, so that $N_{k1} + N_{k2} = N_k$. Individuals in each age-group can be susceptible, infectious asymptomatic, infectious symptomatic, recovered (symptomatic or asymptomatic) and immune. They can can be vaccinated ($j = 1$) or unvaccinated ($j = 0$). Members in age-group i in city k and vaccinated status j are denoted by S_{ijk} for the susceptible classes, A_{ijk} for the infected asymptomatic classes, I_{ijk} for the infected symptomatic classes, R_{ijk} for the recovered symptomatic classes, and RA_{ijk} for the recovered asymptomatic classes, $i = 1, 2$, $j = 0, 1$ and $k = 1, \dots, K$. Following the ideas of [2], three major effects of vaccine are assumed: the vaccine efficacy for susceptibility (the ability of the vaccine to prevent infection) $\mathsf{VE_S}$, the vaccine efficacy for infectiousness (the ability of the vaccine to reduce infectiousness conditioned upon being infected) $\mathsf{VE_I}$, and the vaccine efficacy for pathogenicity (the ability of the vaccine to reduce symptoms conditioned upon being infected) $\mathsf{VE_P}$.

An infectious person becomes either symptomatic or asymptomatic. Infectious symptomatic people have a reduction in their probability of traveling to other cities. Infectious asymptomatic people are still infectious, but have their infectiousness reduced by a predetermined factor m compared to infectious symptomatic people, with $m \in [0, 1]$. The differential equations in each city are described by the following system,

Unvaccinated / Vaccinated

$$\frac{dS_{10k}}{dt} = -\lambda_1 S_{10k} \qquad \frac{dS_{11k}}{dt} = -\lambda_1 \theta S_{11k} \tag{1}$$

$$\frac{dS_{20k}}{dt} = -\lambda_2 S_{20k} \qquad \frac{dS_{21k}}{dt} = -\lambda_2 \theta S_{21k} \tag{2}$$

$$\frac{dA_{10k}}{dt} = \lambda_1(1-\rho)S_{10k} - \gamma A_{10k} \qquad \frac{dA_{11k}}{dt} = \lambda_1(1-\rho\psi)\theta S_{11k} - \gamma A_{11k} \tag{3}$$

$$\frac{dA_{20k}}{dt} = \lambda_2(1-\rho)S_{20k} - \gamma A_{20k} \qquad \frac{dA_{21k}}{dt} = \lambda_2(1-\rho\psi)\theta S_{21k} - \gamma A_{21k} \tag{4}$$

$$\frac{dI_{10k}}{dt} = \lambda_1 \rho S_{10k} - \gamma I_{10k} \qquad \frac{dI_{11k}}{dt} = \lambda_1 \rho\psi\theta S_{11k} - \gamma I_{11k} \tag{5}$$

$$\frac{dI_{20k}}{dt} = \lambda_2 \rho S_{20k} - \gamma I_{20k} \qquad \frac{dI_{21k}}{dt} = \lambda_2 \rho\psi\theta S_{21k} - \gamma I_{21k} \tag{6}$$

$$\frac{dR_{10k}}{dt} = \gamma I_{10k} \qquad \frac{dR_{11k}}{dt} = \gamma I_{11k} \tag{7}$$

$$\frac{dR_{20k}}{dt} = \gamma I_{20k} \qquad \frac{dR_{21k}}{dt} = \gamma I_{21k} \tag{8}$$

$$\frac{dRA_{10k}}{dt} = \gamma A_{10k} \qquad \frac{dRA_{11k}}{dt} = \gamma A_{11k} \tag{9}$$

$$\frac{dRA_{20k}}{dt} = \gamma A_{20k} \qquad \frac{dRA_{21k}}{dt} = \gamma A_{21k} \tag{10}$$

where $\mathsf{VE_S} = 1-\theta$, $\mathsf{VE_I} = 1-\phi$ and $\mathsf{VE_P} = 1-\psi$. The forces of infection for children and adults, respectively, are given by

$$\lambda_1 = \frac{pc_{11}}{N_{k1}}\Big(mA_{10k} + m\phi A_{11k} + I_{10k} + \phi I_{11k}\Big) + \frac{pc_{12}}{N_{k2}}\Big(mA_{20k} + m\phi A_{21k} + I_{20k} + \phi I_{21k}\Big) \tag{11}$$

and

$$\lambda_2 = \frac{pc_{21}}{N_{k1}}\Big(mA_{10k} + m\phi A_{11k} + I_{10k} + \phi I_{11k}\Big) + \frac{pc_{22}}{N_{k2}}\Big(mA_{20k} + m\phi A_{21k} + I_{20k} + \phi I_{21k}\Big). \tag{12}$$

Vaccination

Suppose that we have M doses of vaccine available for age-group i in city k. Ideally, one would like to use all the available vaccine for the susceptible people of age-group i. In practice however, it is very difficult to track susceptible and infectious individuals, especially given that a fraction of the latter are asymptomatic, so a proportion of vaccine is given to people who are already infected or recovered, and it is hence wasted. To take this into account, we use only a fraction f_{ki} of the available vaccine to vaccinate the susceptible individuals of age-group i on day t. This fraction f_{ki} is set to be equal to the fraction of people in age-group i who are still susceptible on day t. Formally, we have for each $k \in \{1, \dots, K\}$ and $i \in \{1, 2\}$,

$$f_{ki} = \frac{S_{i0k}(t)}{N_{ki}}. \tag{13}$$

So for example, if 40% of the children in city i are susceptible on day t, then we will use only 40% of the available vaccine to vaccinate the susceptible individuals of age-group i on day t, and consider that the remaining vaccine is not used.

Calculation of the contact rates

The paper by Wallinga *et al.* [3] served as a basis for our computations for the contact rates between and within the two age-groups. In this section the notation follows that used in [3]. Wallinga *et al.* divided the population into six age classes, a 0-5 class, 6-12 class, 13-19 class, 20-39, 40-59 and 60 or older. They gathered information on the number of conversational partners during a week and the age distribution of these partners. They arranged this information in a matrix M, which they called "social contact matrix". Each entry of the matrix m_{ij} represents the "mean numbers of conversational partners per week in age class i as reported by a participant in class j". We used this matrix to reduce the number of age-groups from six to two by computing weighted averages among the groups.

Let w_i be the projected Dutch population in age-group i, and let w_{tot} be the projected total Dutch population on January 1, 1987.

To obtain the contact rates for our two age-groups, we collapsed three age classes into a single one. Namely, we regrouped all the children into a single class (0-19 years old) and all the adults into a single class ($\geq$ 20). To do this, we started by collapsing two age-groups at a time and apply the algorithm recursively until we have only two age-groups as follows. Assume that we want to collapse age-group i and age-group j into a single age-group. Let this new age-group be the group $\widetilde{ij}$. Furthermore, let $m_{\widetilde{ij},k}$ be the mean number of conversational partners per week in age class $\widetilde{ij}$ as reported by a participant in class k. Then, $m_{\widetilde{ij},k}$ needs to be a weighted average of m_{ik} and m_{jk}, where the weights are given by the fraction of the population in each age-group,

$$m_{\widetilde{ij},k} = \frac{w_i}{w_i + w_j} m_{i,k} + \frac{w_j}{w_i + w_j} m_{j,k}. \tag{14}$$

Analogously, $m_{k,\widetilde{ij}}$ is defined as

$$m_{k,\widetilde{ij}} = \frac{w_i}{w_i + w_j} m_{k,i} + \frac{w_j}{w_i + w_j} m_{k,j}. \tag{15}$$

Finally, $m_{ij,ij}$ is defined also as a weighted average

$$m_{\widetilde{ij},\widetilde{ij}} = \frac{w_i}{w_i + w_j} (m_{ii} + m_{ji}) + \frac{w_j}{w_i + w_j} (m_{jj} + m_{ij}). \tag{16}$$

After repeating this process 4 times, we obtained a 2-by-2 matrix M' of mean number of conversational partners with only two age-groups, given by

$$M' = \begin{bmatrix} 25.3062 & 2.9237 \\ 8.6336 & 32.2160 \end{bmatrix} \tag{17}$$

Then, Wallinga *et al.* defined a "contact matrix" C, where $c_{ij} = m_{ij} w_{tot}/w_i$. Using the same definition, we obtained a matrix C', with $c'_{ij} = m'_{ij} w_{tot}/\tilde{w_i}$ where the weights are now given by the sums of the populations of each collapsed age-group. So, $\tilde{w_1} = w_1 + w_2 + w_3$ and $\tilde{w_2} = w_4 + w_5 + w_6$.

The matrix C' is given by

$$C' = \begin{bmatrix} 93.2188 & 10.7698 \\ 10.7698 & 44.2206 \end{bmatrix}. \tag{18}$$

Finally, we normalize the matrix C' by dividing it by the c'_{11} entry, The matrix $Cnor$ is given by

$$Cnor = \begin{bmatrix} 1 & 0.1155 \\ 0.1155 & 0.4744 \end{bmatrix}. \tag{19}$$

The matrix $Cnor$ is the contact matrix used in the system of ODE's.

Implementation

We adapted Pyevolve [4] so that the evaluation function is set to use our objective function $\mathbf{f}$. We used a crossover rate of 0.8 and a mutation rate of 0.02. We ran the genetic algorithm independently three times. In each of these, we initialized it with 50 chromosomes and let it evolve for 80 generations.

For each of these three individual solutions, we ran 500 epidemics and computed the mean of the attack rate. The optimal solution was selected among the three individual solutions as the one which had the lowest mean attack rate. We then bootstrapped the 500 epidemic results of the optimal solution 1000 times to obtain 95% confidence intervals for the attack rates and the epidemic prevention potential.

We approximate the expected values in equation (**??**) by computing the mean over 20 runs of our model,

$$\mathrm{E}\Big[\sum_{k=1}^{16}\sum_{i=1}^{2}\sum_{j=0}^{1} R_{ijk}\Big] \sim \frac{1}{20}\sum_{l=1}^{20}\Big[\sum_{k=1}^{16}\sum_{i=1}^{2}\sum_{j=0}^{1} R_{ijk}\Big], \tag{20}$$

for minimizing the function $\mathbf{f}$.

Genetic algorithm

A genetic algorithm [5, 6] is a heuristic algorithm that searches for nearly-optimal solutions to a minimization problem by mimicking nature's evolution. The algorithm is initialized with a set of *chromosomes* and evolves in generations. A chromosome is a particular solution to the given problem. Each chromosome is evaluated according to some predetermined fitness function and is assigned a score. In our case, a chromosome is a solution to our optimization problem, and we can think of a gene as a particular control vector. We initialize the genetic algorithm by randomly generating 48 feasible solutions in two ways. First, we generate half of the initial population by randomly (uniformly) selecting numbers between 0 and 1 for each city and age-group in the network, and map the resulting chromosome to the corresponding feasible solution using the transformation T defined in the main text (eq. 9). Second, we create the other half of the chromosomes by randomly selecting a city and an age-group and allocating enough vaccine to cover as many people as possible in this age-group. Then, if vaccine is still available, we select another city and age-group and use the rest of the vaccine to allocate as much as possible in this age-group and so forth until we run out of vaccine.

Sensitivity Analysis

Reproduction number

We performed sensitivity analysis for all the possible scenarios (six different coverages and six different vaccination dates) to evaluate the robustness of our results when the epidemic was assumed to be less transmissible (basic reproduction number $R_0 = 1.2$), when the epidemic was assumed to be more transmissible ($R_0 = 1.8$).

Figures S6 and S7 present the results when we assumed the virus was less transmissible. All three strategies perform better under this scenario. This is expected, as we know from previous studies [1] that for a low R_0 as this one, the threshold in deterministic models for the number of children and adults needed to be vaccinated to bring R_0 below 1 is much lower. Also, it is important to note that when $R_0 = 1.2$, the epidemic is much slower, so that after 250 days (the final time considered for this study) the epidemic has not yet peaked in most of the cities.

Figures S8 and S9 present the results for a more transmissible virus, with $R_0 = 1.8$. Again, as expected, here the three strategies presented performed worse than when $R_0 = 1.5$. The optimal solution always outperforms the other strategies considered, and it is able to reduce the size of the epidemic by more than 40% compared with no vaccination with as few as 4 million doses. Similar to the base case where $R_0 = 1.5$, the biggest difference between the optimal strategy and the children-only pro rata strategy is found when vaccination occurs early, and this

difference tends to decrease as vaccination occurs later. However, this difference is more prominent for $R_0 = 1.8$ than for $R_0 = 1.5$ when vaccination occurs during the first few days of the epidemic and we have more resources available (Fig. S8D-F), suggesting that when the virus is more transmissible the optimal allocation of resources is more important, at least at the beginning of the epidemic.

For the rest of the sensitivity analysis, we picked a single vaccination coverage to perform the analysis. Because the most interesting results (i.e, those where the optimal vaccine distribution showed greatest advantage with respect to the other two strategies considered) were obtained when there were five million doses of vaccine, we concentrated the analysis on this coverage but considered all the six different vaccination dates.

Probability of travel

We next analyzed the sensitivity of our results to the probability of infectious people traveling through the network. To the best of our knowledge, there is no data concerning the probability of travel if a person becomes infected and ill. For this reason, we considered that symptomatic infectious people have a 25% reduction in their probability of traveling when compared to an asymptomatic infectious traveler for the baseline cases. Figures S10 and S11 show the results when symptomatic infectious people have a 10% reduction in their probability of traveling (panel A) or a 75% reduction in their probability of traveling (panel B). A little lower reduction in the probability of traveling yields a slight increase in the attack rates (fig. S10, panel A) and a higher reduction in the probability of traveling yields a decrease in the attack rates. Interestingly, when the probability of travel is reduced by 75%, the optimal strategy is able to prevent 44% of the epidemics (fig. S11, panel B). This is expected, if symptomatic infectious people are traveling less, the epidemic is spreading more slowly and the optimal strategy is able to prevent the epidemics for a longer time. However, the main conclusions are insensitive to this parameter: the optimal strategy outperforms the other two strategies with the major difference seen when vaccination occurs during the first days of the epidemic.

Probability of travel for infected children

In this section we consider the probability of an infected child traveling through the network. Due to the lack of data for this region, we conservatively assumed that infected children will travel with the same probability than infected adults for the baseline cases. Figure S12 shows the attack rates (panel A) and the EPP (panel B) when the optimization was done assuming that infected children will have a 50% less probability of travel than infected adults. The results are very similar to those presented in the main text. The conclusions were not sensitive to this parameter, but, as expected, we obtained a better EPP when the probability of travel for children was reduced by 50%. As before, if fewer infected people (here, children) are traveling the network, the epidemic occurs at a slower pace and the optimal strategy is able to prevent more epidemics.

Vaccination

Figures S13 and S14 present the results when the vaccine efficacies considered are lower than those presented in the main text. We considered two new vaccine efficacy values: the first one assumed that the vaccine efficacies would be one-third of the original values ($\mathbf{VE_S} = 0.13$, $\mathbf{VE_I} = 0.15$, and $\mathbf{VE_P} = 0.25$, results given in panel A) and the second one assumed that vaccine efficacies would be two-thirds of their original values ($\mathbf{VE_S} = 0.27$, $\mathbf{VE_I} = 0.30$, and $\mathbf{VE_P} = 0.50$, results given on panel B). With very low vaccine efficacies (one third of the original values, panel A), the three strategies perform poorly, but the optimal strategy still performs better than the other two strategies for all the dates considered. With low vaccine efficacies (two thirds of the original values, panel B), the results presented in the main text are preserved: the optimal strategy outperforms the other two strategies with a maximum difference attained if vaccination occurs early (11% difference in the attack rate), but this difference quickly declines as vaccination starts later on. We also performed sensitivity analysis with respect to the vaccination timing. Figure S15 presents the results when we assumed that vaccination would be completed in 10 days. Here, the difference in the attack rates between the optimal strategy and the children-only pro rata

strategy is only 5% (panel A). The optimal strategy can contain 36% of the epidemics but only if vaccination starts on day 5.

Optimization when vaccines are only given to children

In this section, we performed the optimization when vaccines can be given only to children. The results are shown in figure S16. The optimal vaccination strategy outperforms the other two strategies. Here, the attack rates are slightly lower than when the optimization is performed over the entire population, but the EPP is very similar. These results suggest that allocating vaccine to the high-transmission groups (in this case children) could be more advantageous than to allocate the vaccine to cities where the epidemic is already under way, or to cities that are well connected.

References

1. Matrajt L, Longini Jr IM (2012) Critical immune and vaccination thresholds for determining multiple influenza epidemic waves. Epidemics 4: 22 - 32.

2. Halloran ME, Struchiner CJ, Longini IM (1997) Study designs for evaluating different efficacy and effectiveness aspects of vaccines. American Journal of Epidemiology 146: 789–803.

3. Wallinga J, Teunis P, Kretzschmar M (2006) Using data on social contacts to estimate age-specific transmission parameters for respiratory-spread infectious agents. American Journal of Epidemiology 164: 936-944.

4. Perone CS (2009) Pyevolve: a Python open-source framework for genetic algorithms. Newsletter ACM SIGEVOlution Volume 4.

5. Goldberg DE (1989) Genetic Algorithms in Search, Optimization and Machine Learning. Kluwer Academic Publishers, Boston, MA.

6. Holland JH (1975) Adaptation in Natural and Artificial Systems. University of Michigan Press, Ann Arbor.
